# Pancrustacean Evolution Illuminated by Taxon-Rich Genomic-Scale Data Sets with an Expanded Remipede Sampling

**DOI:** 10.1093/gbe/evz097

**Published:** 2019-07-04

**Authors:** Jesus Lozano-Fernandez, Mattia Giacomelli, James F Fleming, Albert Chen, Jakob Vinther, Philip Francis Thomsen, Henrik Glenner, Ferran Palero, David A Legg, Thomas M Iliffe, Davide Pisani, Jørgen Olesen

**Affiliations:** 1 School of Biological Sciences, University of Bristol, United Kingdom; 2 School of Earth Sciences, University of Bristol, United Kingdom; 3 Natural History Museum of Denmark, University of Copenhagen, Denmark; 4 Department of Biology, University of Bergen, Norway; 5 Centro de Estudios Avanzados de Blanes (CEAB-CSIC), Blanes, Spain; 6 Faculty of Biology and Environmental Protection, University of Lodz, Poland; 7 Department of Earth, Atmospheric, and Environmental Sciences, University of Manchester, United Kingdom; 8 Department of Marine Biology, Texas A&M University at Galveston; 9 Department of Evolutionary Biology, Ecology and Environmental Sciences, and Biodiversity Research Institute (IRBio), Universitat de Barcelona, Barcelona, Spain; 10 Institute for Advanced Biosciences, Keio University, Tsuruoka, Yamagata, Japan; 11 Department of Biology and Biochemistry, University of Bath, Bath, United Kingdom; 12 Department of Bioscience, University of Aarhus, Aarhus, Denmark

**Keywords:** Pancrustacea, crustacean phylogeny, transcriptomics, Dayhoff recoding, remipedes

## Abstract

The relationships of crustaceans and hexapods (Pancrustacea) have been much discussed and partially elucidated following the emergence of phylogenomic data sets. However, major uncertainties still remain regarding the position of iconic taxa such as Branchiopoda, Copepoda, Remipedia, and Cephalocarida, and the sister group relationship of hexapods. We assembled the most taxon-rich phylogenomic pancrustacean data set to date and analyzed it using a variety of methodological approaches. We prioritized low levels of missing data and found that some clades were consistently recovered independently of the analytical approach used. These include, for example, Oligostraca and Altocrustacea. Substantial support was also found for Allotriocarida, with Remipedia as the sister of Hexapoda (i.e., Labiocarida), and Branchiopoda as the sister of Labiocarida, a clade that we name Athalassocarida (=”nonmarine shrimps”). Within Allotriocarida, Cephalocarida was found as the sister of Athalassocarida. Finally, moderate support was found for Hexanauplia (Copepoda as sister to Thecostraca) in alliance with Malacostraca. Mapping key crustacean tagmosis patterns and developmental characters across the revised phylogeny suggests that the ancestral pancrustacean was relatively short-bodied, with extreme body elongation and anamorphic development emerging later in pancrustacean evolution.

## Introduction

The rapid advancement in DNA sequencing technology has led to major changes in our understanding of crustacean relationships and evolution. Twenty years ago, conflicting morphology-based classification schemes existed, all of which did not recognize that hexapods are nothing but terrestrial crustaceans (see Schram 1986; Walossek 1993; Walossek and Müller 1998b; Martin and Davis 2001). To date, the view that hexapods represent a terrestrial lineage of crustaceans (the Pancrustacea/Tetraconata hypothesis) is nearly universally accepted (see Wägele and Kück 2014 for a contrasting opinion). However, uncertainty remains with reference to the relative relationships within Pancrustacea. Clades such as Copepoda have not yet found a stable position, and much uncertainty still relates to concepts such as “Allotriocarida,” “Multicrustacea,” “Hexanauplia,” and “Communostraca”—table 1 (von Reumont et al. 2012; Oakley et al. 2013; Schwentner et al. 2017, 2018). Perhaps most importantly, it is still unclear what crustacean lineage represent the sister group of the terrestrial hexapods, with recent studies having suggested Xenocarida (Remipedia plus Cephalocarida), Branchiopoda, or Remipedia (e.g., Regier et al. 2010; Lozano-Fernandez et al. 2016; Schwentner et al. 2017, 2018). Finally, attempts to identify morphological synapomorphies for the proposed pancrustacean clades, and attempts at understanding morphological evolution in Pancrustacea (e.g., tagmosis, developmental patterns, or limb morphology) have only just started.

**Table 1:** Overview of Several Proposed Phylogenetic Pancrustacean Clades That Have Been Supported in Phylogenomic Studies Published During the Last 15 Years, Including the Present Work

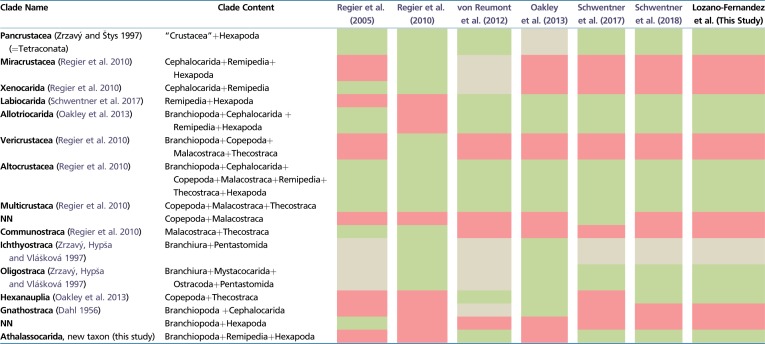

Note.—Green, support; red, lack of support; gray, insufficient data to test concept.

### Morphology-Based Phylogenies

Tagmosis patterns, larval characters, and limb morphology define major crustacean taxa, such as the hyperdiverse Malacostraca (e.g., crabs, shrimps), Branchiopoda (e.g., fairy shrimps), and Thecostraca (e.g., barnacles). Morphology (sperm ultrastructure) even established a surprisingly close relationship between Branchiura and Pentastomida (carp lice and tongue worms; Wingstrand 1972). However, morphology proved to be far from satisfactory at elucidating the relationship between higher level pancrustacean taxa more broadly, and a diversity of contrasting hypotheses have been developed based on alternative interpretations of the morphological evidence. These hypotheses include, among the others: “Maxillopoda” (Copepoda, Thecostraca, Mystacocarida, Branchiura, and Ostracoda—Dahl 1956; Boxshall 1983; Walossek and Müller 1998a, 1998b); “Thoracopoda” (Cephalocarida, Branchiopoda, and Malacostraca—Hessler and Newman 1975); “Entomostraca” (all nonmalacostracans crustaceans—Walossek 1993; Walossek and Müller 1998a); and “Gnathostraca” (Cephalocarida and Branchiopoda—Dahl 1956), the only mentioned hypothesis that has found some support from molecular data (e.g., Oakley et al. 2013; see table 1).

Two crustacean taxa, Cephalocarida and Remipedia were discovered only a few decades ago (Sanders 1955; Yager 1981), and have played a particularly important role in discussions on early crustacean evolution. Cephalocarids are millimeter-sized interstitial sea-bottom crustaceans, with morphological similarities to some of the Cambrian “Orsten” microfossils (Olesen et al. 2011). Accordingly, they were long considered “the best living representation of what the ur-crustacean looked like” (Hessler 1992). Even more unusual are the Remipedia, a lineage of centimeter-sized, multisegmented, predatory, and venomous crustaceans exclusively inhabiting anchialine caves. They were discovered in 1980 (Yager 1981) and their phylogenetic position has long been one of the most debated topics in carcinology. Remipedes long competed with Cephalocarida for the status of the “most morphologically primitive crustaceans” (Yager 1981; Schram 1983; Hessler 1992), until molecular data identified them as closely related to Hexapoda (Regier et al. 2010; von Reumont et al. 2012).

### Molecular-Based Phylogenies

The molecular era of high-level crustacean phylogeny began in the late 1980s when support for the Pancrustacea (or Tetraconata) hypothesis began to emerge (see Zrzavý et al. 1997). Within Pancrustacea the precise sister group of Hexapoda is still debated (see above). Further, unforeseen results that emerged from the analysis of molecular data include the support for new taxa such Oligostraca, a seemingly robust clade including Ichthyostraca, Ostracoda, and Mystacocarida (Zrzavý et al. 1997; Regier et al. 2010; Oakley et al. 2013; Schwentner et al. 2017, 2018), support for Altocrustacea, which includes all pancrustaceans except Oligostraca (Regier et al. 2010; von Reumont et al. 2012; Oakley et al. 2013; Schwentner et al. 2017, 2018), and Allotriocarida, a clade proposed to include Hexapoda, Remipedia, Branchiopoda, and Cephalocarida (von Reumont et al. 2012; Oakley et al. 2013; Schwentner et al. 2017, 2018; table 1).

We have constructed the most taxon-rich pancrustacean phylogenomic data set so far. We improved lineage sampling by adding newly generated transcriptomic data of the pivotal Remipedia, expanded gene sampling, and improved matrix completeness (reduced missing data). We employ a variety of analytical approaches to test the robustness of the results, and interpret the evolution of crustacean tagmosis patterns and developmental characters based on the tree obtained from our analyses.

## Materials and Methods

### Data Acquisition and Transcriptome Assembly

Two molecular matrices were generated by using protein coding genes from 140 species, mostly gathered from Illumina transcriptomes, and largely retrieved from public repositories (supplementary table 1, Supplementary Material online). We generated three new transcriptomes for the following remipede species, *Godzilliognomus frondosus*, *Pleomothra apletocheles*, and *Morlockia williamsi*. For these three species, total RNA extractions were performed using TRIzol Reagent (ThermoFisher Scientific) following the manufacturer’s protocol, with sequencing carried out using Illumina platform, 100 bp read length, paired end reading at the University of Bristol Genomic services, and deposited on NCBI (National Center for Biotechnology Information) under the accession Bioproject number PRJNA507978 (see supplementary table 1, Supplementary Material online). Both the raw sequences downloaded from public repositories and the newly generated ones were processed as follows: Transcriptome assembly was carried out using Trinity version 2.0.3 (Grabherr et al. 2011; Haas et al. 2013) under default parameters and using Trimmomatic (default parameters, as part of the Trinity package) for quality control. To predict the putative proteins from the Trinity assembly results, a previous filter of reduction of redundant isoforms was done by using CD-HIT-EST with a 95% similarity cutoff (Fu et al. 2012). These filtered results were processed in TransDecoder (Haas et al. 2013) in order to identify candidate open reading frames within the transcripts and translate them into proteins.

### Orthology Assignment and Matrix Assembly

We generated two independent molecular data sets based on the transcriptomic data of 140 species. The first supermatrix that was assembled, named “Matrix A,” contained 244 genes and was largely based on the gene sampling of Lozano-Fernandez et al. (2016). Genes in this data set were selected based on being largely single-copy and presenting a slow rate of evolution. The taxonomic sample comprised 125 pancrustaceans, 58 of them being nonhexapods and 67 being hexapods, and 15 outgroups, covering the major groups of interest and being the largest pancrustacean phylogenomic matrix in terms of number of species assembled to date. Through BLAST (Altschul et al. 1990), we acquired the orthologous genes by searching for them on the transcriptomes translated into protein sequences. We used *Daphnia pulex* as the search query due to it being a pancrustacean possessing full coverage of the gene data set. MoSuMa (Lozano-Fernandez et al. 2016; Tanner et al. 2017), a custom Perl script (available at github.com/jairly/MoSuMa_tools/) can be used to relatively quickly and automatically expand a pre-existing phylogenomic data set. For the first step, the best BLAST hits are chosen together with all the sequences with an *e*-value within three orders of magnitude (in order to provide possible alternative orthologs). The minimum *e*-value threshold was set at 10^−20^ for those proteins <150 amino acids, with hits exceeding this being excluded, and was more stringent for proteins >150 amino acids, set at 10^−80^, to exclude false positive orthologs. For each considered protein family, MoSuMa aligns all putative selected orthologs using MUSCLE (Edgar 2004; applying default parameters), to produce gene alignments for each of the 244 genes. Gene trees were inferred for each individual gene alignment using IQ-TREE (Nguyen et al. 2015), applying the model of best fit as assigned by ModelFinder (Kalyaanamoorthy et al. 2017; as part of the IQTree package). For nearly all gene trees, the model LG+I+G4 was best fit. The 244 gene trees were assessed for long branches using a custom Perl script (/github.com/jairly/MoSuMa_tools/blob/master/treecleaner.pl). Sequences producing these long branches were removed from each gene matrix in order to minimize long branch bias in the supermatrix using the criteria defined in Lozano-Fernandez et al. (2016). The gene alignments, thus cleaned of long-branched sequences and putative paralogs, were concatenated using SequenceMatrix v100 (Vaidya et al. 2011). The final resulting supermatrix consists of 57,149 amino acid positions across 140 taxa and with a 75.5% level of completeness. We call this data set “Matrix A.”

We used a different strategy of orthology selection for the second matrix optimized to maximize gene inclusion. This was named “Matrix B,” and was based on the OMA stand-alone software (Roth et al. 2008), that is able to infer and generate clusters of orthologous genes based on a set of transcriptomes using an all-against-all algorithm. In contrast to Matrix A, Matrix B was compiled without attempting to filter out genes based on their expected phylogenetic utility. To limit the computational time during the retrieval of orthogroups, we reduced the transcriptome input used in OMA to 54 transcriptomes that covered most of the diversity of the lineage (species marked in bold in supplementary table 1, Supplementary Material online). The software inferred 116,835 orthologous groups. Nonetheless, many of them had low occupancy across taxa. Therefore, to increase the gene occupancy, we only kept those present in at least 50% of the taxa, ending with a total set of 2,718 orthologous proteins. At this point, we added orthologs using MoSuMa (see above for details). The concatenated supermatrix yielded 1,435,810 amino acid positions. To reduce noise due to potentially misaligned positions, we trimmed this supermatrix using stringent settings in Gblocks v0.91b (Castresana 2000; using the parameters *b*2* *=* *57%, *b*3* *=* *8, *b*4* *=* *10, *b*5* *=* *none), with the aim to reduce missing information, and resulted in a matrix with 53,039 amino acid positions and with a completeness of 72.8%. Matrices A and B (untrimmed and trimmed) are provided in a FigShare repository (10.6084/m9.figshare.8003945).

### Substitution Saturation Analysis

**Fig. 1.**
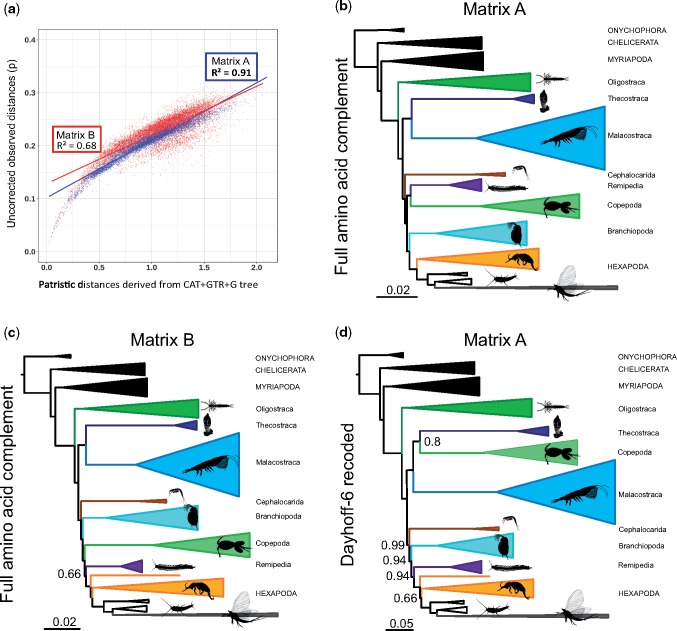
—(*A*) Saturation plots for Matrices A and B showing patristic distances and illustrating that Matrix B has greater level of saturation than Matrix A. (*B–D*) Schematic representation of the Bayesian results of: (*B*) CAT-GTR+G analysis of Matrix A, (*C*) CAT-GTR+G analysis of Matrix B and (*D*) CAT-GTR+G of Matrix A after Dayhoff-6 recoding strategy (outgroups not shown). (*B–D*) Support values represent posterior probabilities and only those <1 are shown. Within Hexapoda, Pterygota are depicted in gray, classically recognized “Entognatha” in orange, and Archaeognatha and Zygentoma in white. Most silhouettes are from Phylopic (phylopic.org/).

APE (Paradis et al. 2004) was used to calculate taxon-to-taxon (i.e., patristic) distances for trees derived using both Matrix A and Matrix B and to estimate saturation plots (fig. 1*A*). To derive the saturation plots, we compared patristic distances from a tree generated using CAT-GTR+G against those from uncorrected observed distances generated for the same matrices in PAUP4.0a (Swofford 2002). When deriving saturation plots the expectation is that uncorrected genetic distances will more strongly underestimate true evolutionary distances as saturation increases (Page and Holmes 1998), because these distances do not account for multiple substitutions. Accordingly, uncorrected observed distances will correlate more poorly with patristic distances derived from a Bayesian tree derived using substitution models, in our case CAT-GTR+G, that allows the estimation of multiple substitution per site. In a saturation plot, the lower the *R*^2^ the greater the saturation of the considered data set.

### Phylogenetic Analyses

We performed phylogenetic analyses using both Maximum Likelihood (ML) and Bayesian Inference. All ML analyses were completed in IQ-TREE (Nguyen et al. 2015) under the LG+I+G4 model. All Bayesian analyses were completed in PhyloBayes MPI v1.5a (Lartillot et al. 2013) under the CAT-GTR+G model. For the IQ-TREE analyses we used LG+I+G4, selected as best fit for Matrix A and B using ModelFinder (Kalyaanamoorthy et al. 2017). CAT-GTR has been shown as the most suitable model for resolving instances of long-branch attraction (Whelan and Halanych 2016); therefore, we assume that CAT-GTR+G is a more complex model than LG+I+G4 that better fits the data. Both Matrix A and Matrix B were analyzed using ML and Bayesian analysis at the amino acid level. However, to assess the potential impact of lineage-specific compositional heterogeneity, we also analyzed Matrix A, the least saturated, after Dayhoff-6 recoding using the CAT-GTR+G model of amino acid substitution (Feuda et al. 2017). Dayhoff-6 recodes the 20 different amino acids into six groups on the basis of their chemical and physical properties. This approach excludes (frequent) replacements between similar amino acids and reduces the effects of saturation and compositional bias (Feuda et al. 2017), bias previously found in pancrustacean phylogenomic matrices which can be partially ameliorated using recoding strategies (Rota-Stabelli et al. 2013).

**Fig. 2.**
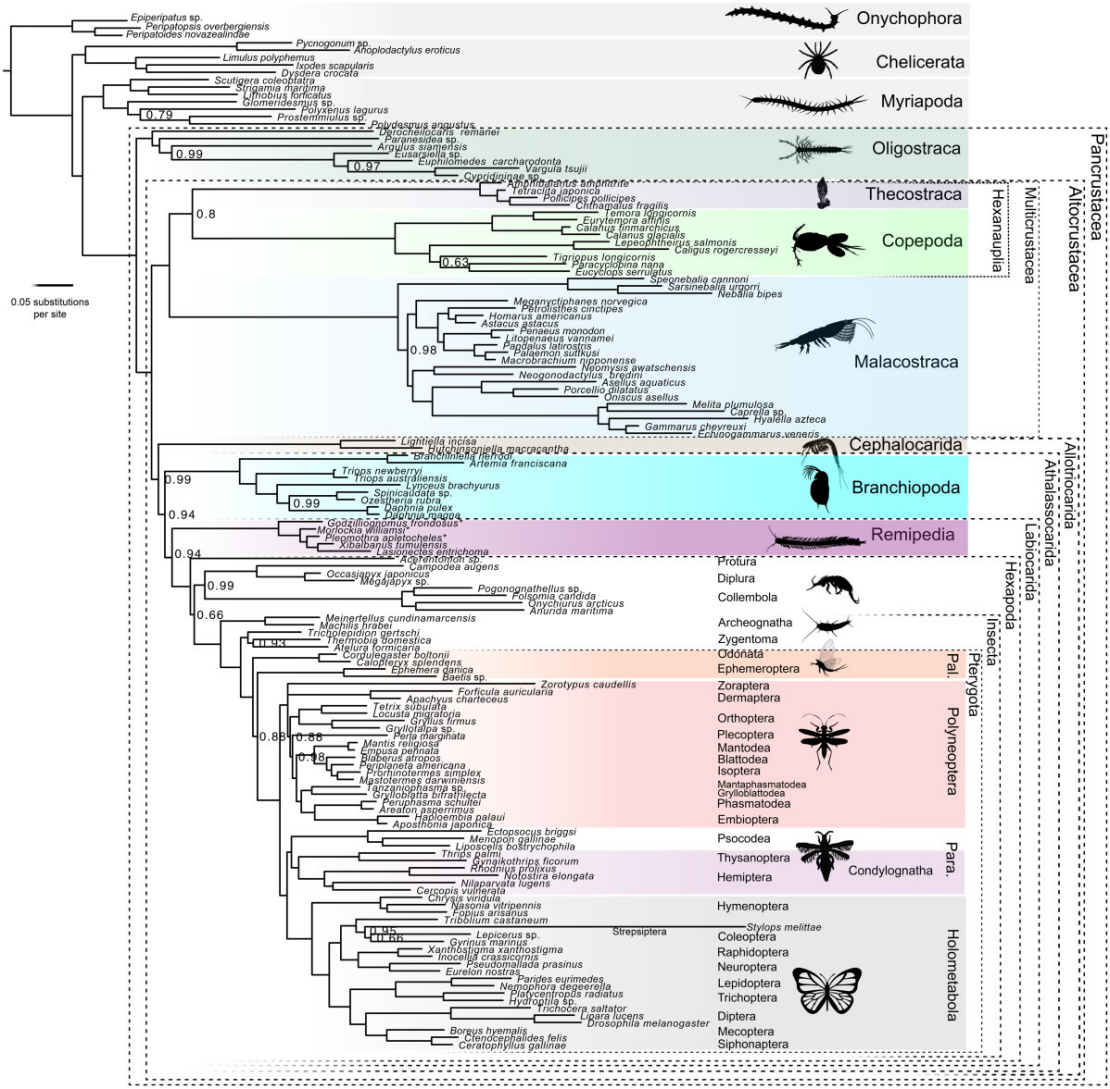
—Phylogenetic tree derived from the CAT-GTR+G analysis of the Matrix A recoded version under Dayhoff-6. Newly sequenced transcriptomes are marked with an asterisk. Burnin = 2,500, Total Cycles=10,000, subsampling frequency=10, Maxdif=0.22, Minimal effective size=64. Support values represent posterior probabilities and only those <1 are shown. Para., Paraneoptera; Pal., Paleoptera.

Two independent runs were completed for all PhyloBayes analyses. Convergence was tested using the maximum difference in the bipartitions of the chains, as reported by *bpcomp* program in the PhyloBayes package. A further test of convergence was carried out using *tracecomp* (also part of PhyloBayes), where we evaluated the effective sample sizes and relative differences for all parameters. These are the results of the three CAT-GTR+G Phylobayes: Matrix A (fig. 1*B*): Burnin = 2,500, Total Cycles = 10,000, subsampling frequency = 20, Maxdif = 1.00, Minimal effective size = 10; Matrix B (fig. 1*C*): Burnin = 2,500, Total Cycles = 10,000, subsampling frequency = 20, Maxdif = 0.30, Minimal effective size = 30; Matrix A Dayhoff-6 recoded (figs. 1*D* and 2): Burnin = 2,500, Total Cycles = 10,000, subsampling frequency = 20, Maxdif = 0.22; Minimal effective size = 64. Support in our Bayesian trees represents Posterior Probabilities. Support values in the ML trees are bootstrap proportions. Bootstrap analyses in IQTree used 1,000 replicates and the ultrafast inference method (Minh et al. 2013).

### Character Mapping

**Fig. 3.**
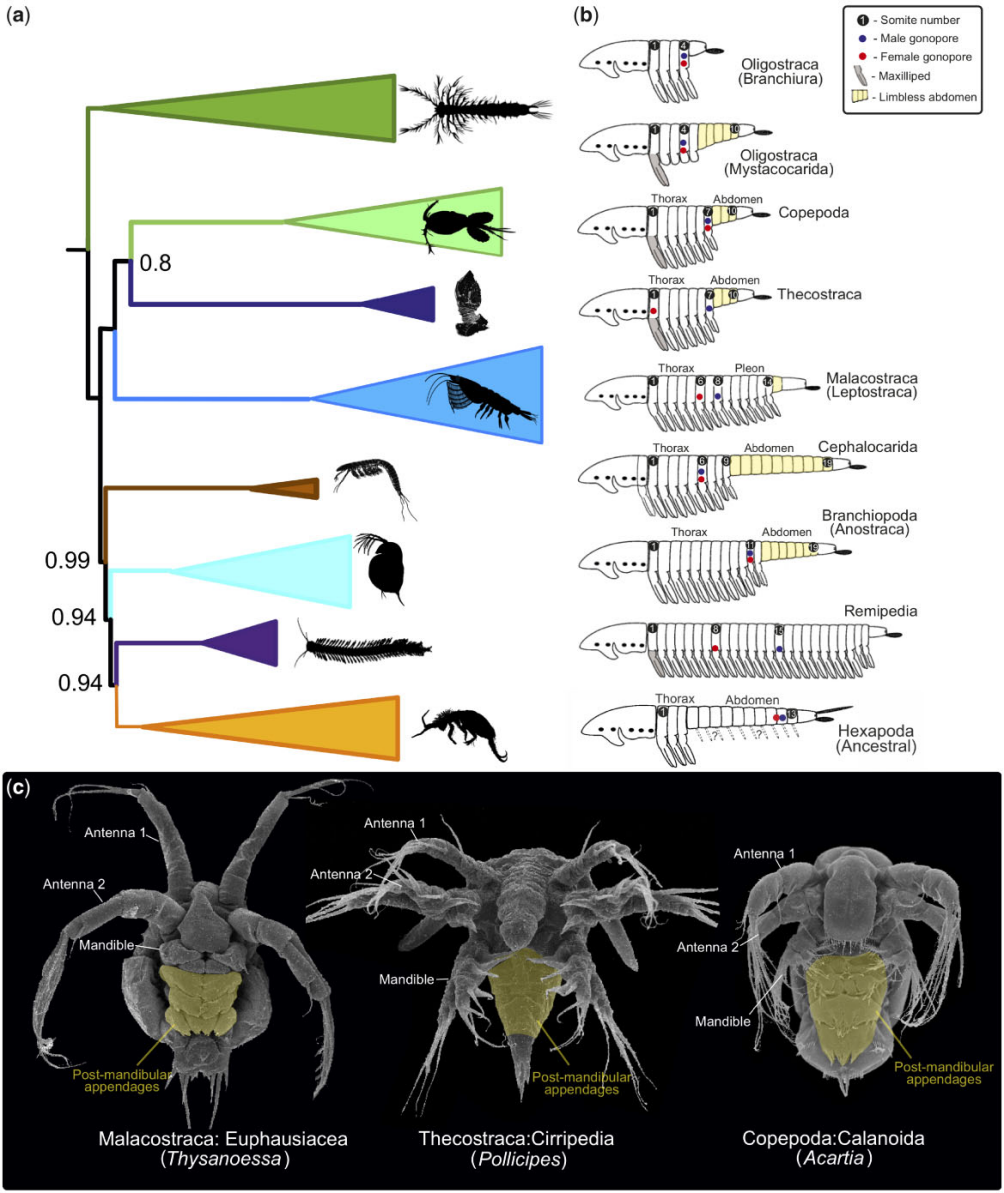
—Some classical crustacean characters relating to tagmosis and development mapped on a summarized version of the most robust phylogeny. (*A*) Schematic representation of CAT-GTR+G phylogeny of Matrix A after Dayhoff-6 recoding strategy. (*B*) Tagmosis patterns and gonopore positions in major taxa of Pancrustacea (figure modified from Walossek and Müller 1998a and supplemented from Boxshall 1983; Olesen 2001; Grimaldi and Engel 2005). (*C*) Nauplii/metanauplii of Malacostraca, Thecostraca, and Copepoda with delayed development of postmandibular limbs during naupliar sequence, which is a putative synapomorphy for Multicrustacea (see Discussion) (figure modified from Akther et al. [2015] [*Thysanoessa*] and Olesen 2018 [*Pollicipes* and *Acartia*]).

Some classical crustacean characters relating to tagmosis and development were mapped on a summarized version of the less saturated matrix A and using the most complex model (CAT-GTR+G) after Dayhoff-6 recoding strategy (fig. 3*A* and *B*). The variation within Pancrustacea in body length and gonopore positions is enormous, but it has long been known that some higher level taxa display rather fixed patterns. These patterns were summarized by Boxshall (1983) and Walossek and Müller (1998a), which were used as the basis for the information in figure 3*B* (supplemented by Olesen 2001; Grimaldi and Engel 2005).

### Data and Software Availability

The OMA-predicted orthogroups, amino acid matrices and phylogenetic trees are available in a FigShare repository (10.6084/m9.figshare.8003945). The transcriptomes generated as part of our study are available in the NCBI Sequence Read Archive with BioProject number PRJNA507978. Individual SRA numbers for the raw read data of each species are listed in supplementary table 1, Supplementary Material online.

## Results

### Overview

We present a phylogenomic investigation of Pancrustacea based on two new molecular matrices derived using transcriptomic and genomic data from 140 species, 125 of them being pancrustaceans. Our data sets include representatives of all pancrustacean classes as well as covering most hexapod orders. We expanded the taxon sampling adding more copepods, branchiopods, and particularly remipedes. For the latter, we have added transcriptomes for three new families, thereby including a total of five different remipede families in our data set. We focused on reducing missing data, and particularly on results of analyses that attempted to minimize sequence saturation and compositional heterogeneity (e.g., Feuda et al. 2017; Laumer 2018).

### Molecular Data Sets and Model Selection

To test the robustness of inferred phylogenetic relationships, we generated two independent data sets using different strategies of orthology selection designed to achieve different kinds of optimizations. The first data set, named “Matrix A,” was based on our legacy data set (Rota-Stabelli et al. 2013; Lozano-Fernandez et al. 2016) and is represented by a super-alignment including 57,149 amino acid positions (75.5% complete) and 244 loci. The genes present in Matrix A were selected to maximize inclusion of known single-copy genes (to minimize the negative effects of hidden paralogy), and slowly evolving, informational genes such as ribosomal proteins (to reduce the negative impact of saturation-dependent tree reconstruction artifacts, like Long Branch Attraction [LBA]) (Sperling et al. 2009; Pisani et al. 2015). The second strategy, resulting in the generation of “Matrix B,” was based on maximizing gene inclusion. Matrix B was constructed using the OMA stand-alone software (Roth et al. 2008) to de novo identify orthologs from a set of transcriptomes. Using this approach, we generated a new set of 3,139 loci based on the OMA-selected genes that were present in at least in 50% of the taxa in our data set. The retained high-occupancy genes were concatenated and posteriorly trimmed stringently to remove poorly aligned positions in a final supermatrix representing 53,039 amino acid positions (72.8% complete); see Materials and Methods section for details. Based on previous results, which suggest that pancrustacean phylogenies might be prone to LBA artifacts (Schwentner et al. 2017), we used saturation plots to compare substitutional-saturation levels between Matrix A and B, as increased substitutional-saturation is directly linked to the emergence of tree reconstruction artifacts (Page and Holmes 1998; Philippe et al. 2011; Pisani et al. 2015). Our saturation plots (fig. 1*A*) indicated that Matrix A, originally designed to include slowly evolving genes, is in fact less saturated (*R*^2^ = 0.91) than Matrix B (*R*^2^ = 0.68), which was generated from a large set of orthologs that was not filtered to remove genes with high rates of evolution. This result is not surprising given that the strategy followed to derive Matrix B sampled orthologous homogeneously from the considered transcriptomes, without filtering genes with high evolutionary rate out. Substitutional saturation is not the only factor that can negatively affect phylogenetic analyses. Compositional heterogeneity (across both sites and lineages) can also lead to the recovery of artifactual phylogenies (e.g., Feuda et al. 2017). Accordingly, both Matrix A and Matrix B were analyzed using the compositionally site-heterogeneous CAT-GTR+G model in a Bayesian framework (Lartillot and Philippe 2004). Furthermore, as substitutional saturation and compositional heterogeneity can be further reduced using recoding strategies (Feuda et al. 2017 and reviewed in Laumer 2018), we analyzed Matrix A (the least saturated data set) under CAT-GTR+G after Dayhoff-6 recoding (see Feuda et al. 2017 for details). The CAT-GTR+G analysis of the amino acid version of Matrix B converged well, however the amino acid version of Matrix A (fig. 1*B* and supplementary fig. 2*A*, Supplementary Material online) did not converge. Nonetheless, the Dayhoff-6 recoded analysis of Matrix A also converged. This might suggest some compositional problem with this data set. Accordingly, we shall mostly discuss CAT-GTR+G results for Matrix B and for the Dayhoff recoded version of Matrix A (with results of the amino acid version of Matrix A reported for completeness only). Matrices A and B were also analyzed using ML under the LG+I+G4 model using IQ-TREE (Nguyen et al. 2015). LG+I+G4 was used for these analyses as it emerged as the best-fitting model among the set of predefined empirical GTR matrices available in IQ-TREE according to ModelFinder (Kalyaanamoorthy et al. 2017).

### Phylogenetic Patterns in Pancrustacea

All CAT-GTR+G analyses of our amino acid data sets (including the unconverged analyses of Matrix A) recover accepted arthropod relationships, with Pancrustacea being sister to Myriapoda (Mandibulata), and this clade as the sister group of Chelicerata (fig. 1*B–D* andsupplementary fig. 1, Supplementary Material online). Under CAT-GTR+G both matrices support the monophyly of all crustacean classes, with Oligostraca emerging as the sister of Altocrustacea (fig. 1*B* and *C*). Similarly, both matrices suggest that Altocrustacea is composed of two clades, the first consisting of a sister group relationship between Malacostraca and Thecostraca, and the second consisting of Hexapoda, Remipedia, Branchiopoda, Cephalocarida, and Copepoda (fig. 1*B* and *C*). This clade was also obtained by Rota-Stabelli et al. (2013) who referred to it as “Clade A.” Essentially, this clade can be described as a modified version of Allotriocarida, to include also Copepoda. Cephalocarida appears as the earliest diverging lineage within “Clade A.” The sister group relationship of Hexapoda differs between matrices, with the unconverged analysis of Matrix A supporting Branchiopoda as sister of Hexapoda, and the converged analysis of Matrix B supporting Remipedia (fig. 1*C*). CAT-GTR+G is able to model site-specific compositional heterogeneity, but lineage-specific compositional heterogeneity can potentially affect phylogenetic results negatively (Feuda et al. 2017), and different arthropod lineages are known to be affected by strong compositional and synonymous codon usage biases (Rota-Stabelli et al. 2013). CAT-GTR+G analyses of the Dayhoff-6 recoded version of Matrix A, interestingly, found a topology more similar to that obtained from the converged analyses of Matrix B than from the unconverged analyses of Matrix A. This suggests that the results of the unconverged CAT-GTR+G analysis of the amino acid version of Matrix A are likely to represent a suboptimal topology and should not be trusted when in disagreement with results from other analyses. Notably, the Dayhoff-6 analysis of Matrix A supported a monophyletic Allotriocarida (to the exclusion of Copepoda), with Remipedia showing a highly supported sister group relationship with Hexapoda (Labiocarida—Schwentner et al. 2017). In this tree, Branchiopoda representing the sister group of Labiocarida (figs. 1*D* and 2). We propose the name Athalassocarida for the Labiocarida plus Branchiopoda clade (derived from “Athalasso” [Greek: nonmarine] and “carida” [Greek: prawn]), thereby referring to a grouping of pancrustaceans where all extant members either live in nonmarine settings or reverted to a marine life-style secondarily. The Dayhoff-6 analysis of Matrix A also found a sister group relationship between Copepoda and Thecostraca (the Hexanauplia hypothesis), albeit with moderate support (PP = 0.8).

When both data sets are analyzed using ML under the less fitting (with reference to CAT-GTR+G) LG+I+G4 model (supplementary fig. 1*A* and *B*, Supplementary Material online), a tree is obtained where Remipedia plus Cephalocarida (i.e., Xenocarida) is the sister of Hexapoda. This result, first obtained by Regier et al. (2010) was suggested to be artifactual by Rota-Stabelli et al. (2013) and Schwentner et al. (2017). Our analyses, finding this clade only when using less fitting models, reinforce the view that this clade is most likely an artifact. Furthermore, ML analysis of Matrix A did not find support for Allotriocarida, while that of Matrix B only found ambiguous support for this group. Both analyses found strong support for Copepoda as the sister of Malacostraca plus Thecostraca.

### Phylogenetic Patterns within Hexapoda

All analyses recover the monophyly of Pterygota, Polyneoptera, and Holometabola, and within the latter clade, all analyses recover Mecoptera as the sister lineage of Siphonaptera, and this clade as the sister group of Diptera (fig. 2 andsupplementary fig. 2*A–D*, Supplementary Material online). Whereas the CAT-GTR+G (supplementary fig. 2*B*, Supplementary Material online) and ML analyses using LG+I+G4 of Matrix B (supplementary fig. 2*D*, Supplementary Material online) yield Strepsiptera as the sister lineage of Coleoptera, the analyses of Matrix A retrieve Strepsiptera within Coleoptera (fig. 2 andsupplementary fig. 2*A* and *C*, Supplementary Material online). The monophyly of Paraneoptera (fig. 2 andsupplementary fig. 2*A, C*, and *D*, Supplementary Material online) and Condylognatha (fig. 2 andsupplementary fig. 2*A–C*, Supplementary Material online) is supported in most analyses. Within Polyneoptera, all analyses recover a monophyletic Dictyoptera, a clade composed by Mantodea and Blattodea (which also contains Isoptera), and sister group relationships between Phasmatodea and Embioptera, and between Mantophasmatodea and Grylloblattodea (fig. 2 andsupplementary fig. 2*A–D*, Supplementary Material online). The phylogenetic position of Zoraptera, Plecoptera, Dermaptera, and Orthoptera are more contentious due to topological variability between different analyses. None of our CAT-GTR+G analyses yielded a monophyletic Paleoptera, with Ephemeroptera being more closely related with Neopterans than Odonata (fig. 2 andsupplementary fig. 2*A* and *B*, Supplementary Material online). However, the ML analyses of Matrix A and B retrieve the monophyly of Paleoptera (supplementary fig. 2*C* and *D*, Supplementary Material online). Archaeognatha is recovered as the earliest-diverging insect group in all analyses (fig. 2 andsupplementary fig. 2*A–D*, Supplementary Material online). Within the noninsect hexapods, Protura appears as the earliest-diverging clade within Hexapoda in the CAT-GTR+G analyses of Matrix B and the Dayhoff recoded version of Matrix A (supplementary fig. 2*B*, Supplementary Material online and fig. 2, respectively), with Collembola being the sister group of Diplura. The unconverged CAT-GTR+G analysis of Matrix A and the LG+I+G4 analyses of Matrix A and B yielded monophyly of the noninsect hexapods, classically known as Entognatha, in the first instance with a sister group relationship between Protura and Diplura, and in the LG+I+G4 analyses with Protura as sister to Collembola, a lineage known as Ellipura (supplementary fig. 2*A, C*, and *D*, Supplementary Material online). As the latter clades emerge from unconverged analyses and from ML analyses that used less fitting models, we suggest these results are likely to be artifactual.

## Discussion

### Pancrustacean Relationships

Our analyses found a number of major clades to show up consistently (figs. 1*B–D* and 2). All analyses supported a basal division of Pancrustacea into two clades: Oligostraca and Altocrustacea. Oligostraca is a surprising assemblage of mostly short-bodied crustaceans (Ostracoda, Mystacocarida, Branchiura, and Pentastomida), suggested initially by Zrzavý et al. (1997), based primarily on gene expression data and supported repeatedly since (Regier et al. 2010; Oakley et al. 2013; Rota-Stabelli et al. 2013; Schwentner et al. 2017, 2018). Within Oligostraca, we find some evidence for a paraphyletic Ostracoda with Myodocopida being closer to Branchiura than to Podocopida, but this is based on a small taxon sample, and conflicts with Oakley et al. (2013) who found a monophyletic Ostracoda under certain analytical parameters. All other pancrustaceans, including hexapods, group in the Altocrustacea, a clade suggested by Regier et al. (2010) and supported by subsequent studies (von Reumont et al. 2012; Oakley et al. 2013; Rota-Stabelli et al. 2013; Schwentner et al. 2017, 2018; table 1).

We increased sampling of remipedes by adding three newly generated transcriptomes, for a total of five different families. The converged CAT-GTR+G analyses of Matrix B and the converged CAT-GTR+G analysis of Matrix A (with Dayhoff recoding) find support for Remipedia as the sister group of Hexapoda, whereas the unconverged CAT-GTR+G analysis of Matrix A as well as ML analyses of both matrices (that used less fitting models) do not find Remipedia as the exclusive sister to Hexapoda. Overall, we can only conclude that the presented evidence suggests Remipedia as the most likely sister group of Hexapoda. In agreement with previous studies, we suggest that Xenocarida (Remipedia+Cephalocarida) is an attraction artifact, and contrary to Glenner et al. (2006) or Lozano-Fernandez et al. (2016) we conclude that Branchiopoda is unlikely to represent the sister group of Hexapoda. A close relationship between Remipedia and Hexapoda has been suggested a number of times before (von Reumont et al. 2012; Oakley et al. 2013; Schwentner et al. 2017, 2018). This clade is possibly characterized by the presence of a “lower lip” (labium in hexapods; Wägele and Kück 2014)—a character that has been used to name this clade Labiocarida (Schwentner et al. 2017). The branching pattern of the serotonin-expressing neurons (Stemme et al. 2013), as well as features of central nervous system organization (although these are possibly also shared with malacostracans), such as pathways of olfactory glomeruli to the protocerebrum, and fan-shaped midline neuropils (Strausfeld and Andrew 2011; Strausfeld 2012) might constitute further apomorphies of this clade. Branchiopoda is most likely the sister group of Labiocarida as in Schwentner et al. (2017). All three taxa (remipedes, branchiopods, and hexapods) are either nonmarine or have secondarily reverted to marine environments so we suggest the name Athalassocarida in recognition of this. The most likely position of Cephalocarida is inferred to be as sister to Athalassocarida together forming Allotriocarida (but see below).

An additional result of the present work is that a number of traditional class-level groups within Pancrustacea were fully supported in all analyses. However, our taxon sampling only allows for limited discussion of the internal branching patterns of these clades. Within Branchiopoda, the branching pattern follows the generally accepted view (Richter et al. 2007; Olesen 2009; Olesen and Richter 2013; Schwentner et al. 2018). Higher level groupings such as Anostraca, Phyllopoda, and Diplostraca are supported. Within Diplostraca, Laevicaudata and Onychocaudata are sister taxa. Malacostraca are relatively well represented in our data set, and we found Leptostraca as sister to the remaining Malacostraca—in accordance with conventional views (Richter and Scholtz 2001). Decapods constitute a monophyletic lineage with the euphausiacean *Meganyctiphanes norvegica* as its sister lineage, again in accordance with conventional views (Martin and Davis 2001), but in conflict with Shen et al. (2015), who, based on mitochondrial data found Euphausiacea and Dendrobranchiata as sister taxa. Peracarida was retrieved in the analyses of Matrix B, whereas none of the analyses of Matrix A found this clade due to a diverging placement of *Neomysis*. However, it should be noted that our study only included amphipods, isopods, and one mysid. Schwentner et al. (2018) found a monophyletic Peracarida, but the peracarid question clearly needs more attention. In our analysis the single stomatopod included is not near the base of Malacostraca according to Matrix A. Instead it appears close to a Eucarida clade, as sister to the only included mysid. However, in the CAT-GTR+G analysis of Matrix B stomatopods are resolved in accordance with classic views as the next branch after Leptostraca (Richter and Scholtz 2001; Schwentner et al. 2018). The five included species of Remipedia show a phylogenetic topology partly incongruent with Hoenemann et al. (2013). In the present study and in Hoenemann et al. (2013)*Godzilliognomus* constitutes an early branch, which may suggest that the relatively low number of somites in *Godzilliognomus* is closer to the ancestral remipede pattern than that seen in the longer-bodied *Xibalbanus*. The relationships between the remaining four included species are rather different from previous results (Hoenemann et al. 2013), which suggests that Remipedia phylogeny would benefit from a reanalysis using a targeted molecular data set.

Major divisions in insects, such as Pteryogota, Holometabola, and Polyneoptera were recovered in accordance with previous molecularly based phylogenetic studies (Misof et al. 2014; Rainford et al. 2014; Wiens et al. 2015; Song et al. 2016). Within Holometabola, we found Mecoptera as sister group to Siphonaptera, as in Rainford et al. (2014) and Wiens et al. (2015). The Bayesian and ML analyses of Matrix B yielded Strepsiptera as sister group to Coleoptera, in agreement with current consensus (Niehuis et al. 2012). However, converged analyses of Matrix A under Dayhoff recoding retrieved Strepsiptera within Coleoptera. As only one taxon was included in our analyses, we suggest that the long-standing debate on the position of Strepsiptera might benefit from increased taxon sampling. Paraneoptera and Condylognatha are supported in most analyses (as in Rainford et al. 2014; Wiens et al. 2015). The most contentious phylogenetic resolution is found within Polyneoptera, in clades such as Zoraptera, Plecoptera, Dermaptera, and Orthoptera, which are possibly due to low taxon representation. Our CAT-GTR+G analyses surprisingly did not retrieve the monophyly of Paleoptera. Previous investigations have found that this particular clade is highly sensitive to data and method choice (Thomas et al. 2013). As we only included four species, our results should be taken with caution. We recover Archaeognatha as the earliest diverging lineage within insects and Zygentoma as the sister group to the remaining insects, as in most previous phylogenomic analyses (Misof et al. 2014; Wiens et al. 2015; Song et al. 2016). Regarding what has classically been recognized as Entognatha (Protura, Diplura, and Collembola), the analysis using the data, model, and recoding strategy that should minimize the appearance of tree reconstruction artifacts (fig. 2) did not recover its monophyly and rather suggest Protura as the earliest divergent Hexapoda clade. In contrast, other analyses recover a monophyletic Entognatha with some variable intrinsic sister group relationships, either with an alliance between Protura and Diplura (as in Rainford et al. 2014; Wiens et al. 2015) or with the monophyly of Ellipura (Protura and Collembola).

### Major Conflicts and Unsupported Concepts

Although we have assembled comprehensive molecular matrices and used several methods to account for different methodological biases, the phylogenetic position of several taxa, such as Copepoda, are not yet convincingly resolved. Strong support for Allotriocarida is found when Matrix A is analyzed under Dayhoff recoding (with some moderate support found for Copepoda + Thecostraca—collectively known as Hexanauplia). However, Copepoda is recovered as a member of Allotriocarida in the CAT-GTR+G analyses of Matrix B (see also Rota-Stabelli et al. 2013), and as the sister of Malacostraca plus Thecostraca in the ML analyses performed under LG+I+G4. Hence, based on the data presented in this work it is not possible to confidently support the relative relationships of Copepoda, Malacostraca, and Thecostraca. Nonetheless, the phylogenetic analyses performed using the model, data set and recoding strategy that should minimize the appearance of tree reconstruction artifacts (fig. 2) support the exclusion of Copepoda from Allotriocarida and provide moderate support for Hexanauplia within Multicrustacea.

A large number of concepts in pancrustacean phylogeny have been suggested during the preceding decades (see Introduction), many of which are not supported by the present work. Of these, Maxillopoda, which has perhaps been the most persistently discussed, did not receive support in any of our analyses. The same applies to Entomostraca. Some clades based on molecular grounds such as Miracrustacea or Vericrustacea (Regier et al. 2010) were only retrieved when using LG+I+G4 (supplementary fig. 2*C* and *D*, Supplementary Material online), and we suggest that these clades are likely artifactual (see also Rota-Stabelli et al. 2013; Schwentner et al. 2017; see table 1).

### Evolution of Crustacean Tagmosis and Developmental Patterns

It is striking that the topology shown in figure 2 has never been suggested based on morphology (e.g., Wolfe and Hegna 2014). Morphology, though, still has its place in understanding high-level crustacean evolution since one of the goals of evolutionary biology is elucidating phenotypic evolution. Here, we map some classical characters relating to tagmosis patterns and development in an attempt to understand general evolutionary patterns in crustaceans.

Tagmosis patterns in crustaceans are well-known to include more variation than in Hexapoda. Much diversity is seen in body length, number of appendages, division in appendage series into functional units, and the position of gonopores or penial structures (e.g., Boxshall 1983; Walossek and Müller 1998a; Olesen 2001). Tagmosis patterns have traditionally been important for recognizing at least one classical group within Crustacea, the Malacostraca, with its largely constant division into an 8 (thorax) plus 6(7) (pleon) pattern (Calman 1909). In addition, Maxillopoda was largely defined on the basis of tagmosis patterns with similarities in total somite number (10 or 11) and thorax/abdomen division (7 + 4 somites; Boxshall 1983; Walossek and Müller 1998a, 1998b; Olesen 2001). In figure 3, we have superimposed various tagmosis features over the topology of figure 2. Using this, we briefly address the following questions: 1) Is the abdomen in different crustacean subgroups homologous?; 2) Is there any phylogenetic pattern in total body length?; and 3) Is there a pattern in the position of gonopores?

An abdomen in Crustacea is normally defined as a posterior body part devoid of limbs and is present in certain taxa within Oligostraca (e.g., Mystacocarida), Copepoda, Thecostraca, Malacostraca (Leptostraca), Cephalocarida, Branchiopoda, and in Hexapoda (fig. 3*B*). We find largely no phylogenetic pattern in the absence/presence of an abdomen within Pancrustacea and consider this characteristic prone to convergence. Exceptions are Thecostraca and Copepoda where the abdomen includes somites posterior to somite 7, which, taking into account the sister group relationships shown in (figs. 2 and 3*A*), may actually constitute an apomorphy for Hexanauplia (see Boxshall 1983; Walossek and Müller 1998b).

Much variation is seen in total body length in nonhexapod Pancrustacea, with branchiurans (carp lice), some ostracods (mussel shrimps), and some cladocerans (water fleas) being the shortest (down to four postcephalic somites), and remipedes and some branchiopods (among spinicaudatans and notostracans) being the longest (30+ postcephalic somites; Boxshall 1983; Olesen 2001). The large variation makes evolutionary conclusions difficult, but superimposing body length over the topology shown in figure 2 reveals some likely evolutionary patterns challenging commonly held beliefs, for example, that the “ur-crustacean” was “many-segmented” (e.g., Hessler and Newman 1975). Among the generally short-bodied oligostracans, there is significant variation in body length with the ultrashort branchiurans at one end of the spectrum (four somites) and the mystacocarids at the other end (ten postcephalic somites plus telson). The presence of ten postcephalic somites in Thecostraca, Copepoda, and Mystacocarida was considered a key feature uniting “Maxillopoda” (Boxshall 1983; Walossek and Müller 1998b). If the ten somite pattern in these three taxa is homologous, then this number could be considered the ancestral pancrustacean (rather than maxillopodan) pattern, followed by shortening or multiplication in other clades. Analyses using ancestral character state reconstruction should be used to test this conjecture. Outgroup comparison points in the same direction. Within the fossil record, the closest relatives to crown Pancrustacea are among the uniquely preserved Cambrian “Orsten” microarthropods (Walossek and Müller 1990). A number of these fossils have been identified as likely members of the “crustacean” stem lineage (=pancrustacean stem lineage). They are all relatively short-bodied, for example *Martinssonia*, *Oelandocaris*, and *Phosphatocopina*, which have less than ten post “cephalic” somites (respectively, Müller and Walossek 1988; Maas et al. 2003; Stein et al. 2005). In contrast, it is noteworthy that all the long-bodied crustaceans are in the Allotriocarida clade, and that two of these, Cephalocarida and Branchiopoda (Anostraca), share the exact same number of postcephalic somites: 19. This may be indicative that the ancestral number of somites in Allotriocarida increased in Remipedia and within Branchiopoda (some notostracans and spinicaudatans) and got reduced in hexapods. This conjecture should be tested using ancestral character state reconstruction methods.

Gonopore position certainly holds important phylogenetic information for some crustaceans. Nearly all malacostracans have a similar position of the gonopores, associated with thoracic somite 6 in females, and with somite 8 in males. In Thecostraca and Tantulocarida, the female gonopores are uniquely located on the first thoracic somite (Huys et al. 1993). Mapping features relating to gonopore position over the CAT-GTR+G results of the recoded version of Matrix A (fig. 3) reveals no clear evolutionary patterns. In Malacostraca and Thecostraca, the female and male gonopores are placed on two different somites, the female anteriorly and the male posteriorly. This separation in position of the female and male gonopore tentatively qualifies as a synapomorphy of Communostraca (fig. 1*B* and *C*;supplementary fig. 1*A* and *B*, Supplementary Material online). Elsewhere such a separation is seen only in Remipedia. In some oligostracans, the gonopores are placed at the fourth (Branchiura, Mystacocarida) or third somite (Ostracoda; Boxshall 1983), which, in light of the monophyly of Oligostraca may have phylogenetic significance.

Developmental patterns in the nonhexapod part of Pancrustacea are diverse and includes gradual (anamorphic), metamorphic, and epimorphic (“brooding”) development, often combining several types in a single sequence (Walossek 1993; Martin et al. 2014; Olesen 2018). Here, we discuss some selected aspects of crustacean development based on the topology shown in (fig. 3). Often copepods and thecostracans start their development with a sequence of six naupliar stages, followed by a metamorphosis into a new type of larva/juvenile with more appendages (copepodite or cyprid). Oakley et al. (2013) found, as we do under certain parameters, support for a close relationship between Copepoda and Thecostraca and termed the clade “Hexanauplia,” thereby referring to the presence of six nauplii in the naupliar phase. However, under other analytical parameters, we retrieve Thecostraca and Malacostraca as sister taxa (conflicting with the Hexanauplia concept), so we use this opportunity to elaborate and expand on the suggestion by Oakley et al. (2013). As implied by Oakley et al. (2013), postmandibular limbs are either largely absent or are present only as buds during the early development of both Copepoda and Thecostraca (fig. 3*B* and *C*), resulting in a naupliar phase where the only active limbs are the anterior naupliar appendages (antennae 1, 2, and mandibles; see Müller and Walossek 1988; Walossek and Müller 1998b). Comparing malacostracan development with that of Copepoda and Thecostraca is of relevance, but this undertaking is challenging due to an enormous diversity in the development of malacostracans, spanning from rather anamorphic development with free nauplii in krill and dendrobranchiatan shrimps to direct or epimorphic development in leptostracans and peracarids, and not least due to the many spectacular larval types seen in decapods (Martin et al. 2014). Krill and dendrobranchiatan shrimps, uniquely among malacostracans, have free nauplii in the early part of their development, making them likely candidates for a close resemblance to the ancestral malacostracan ground pattern (see Scholtz 2000; Akther et al. 2015) and therefore obvious choices for comparison with nonmalacostracans. Dendrobranchiatan shrimps usually pass through a naupliar phase with five to six stages with postmandibular limbs present only as limb buds (as in many copepods), followed by an abrupt shift into a larval phase with more active appendages (protozoea) (e.g., Chio and Hong 2001; Martin et al. 2014). During krill development there is also a naupliar phase (but of shorter duration) either without (orthonauplii) or with (metanauplius) postmandibular limb buds prior to an abrupt morphological shift into a calyptopis larvae with more active appendages (Suh et al. 1993; Akther et al. 2015) We find that Euphausiacea and Dendrobranchiata (Decapoda) display a suppression of postmandibular limbs during early development (Martin et al. 2014; Olesen 2018) comparable to that seen in Copepoda and Thecostraca (fig. 3*C*). Such limb suppression in early naupliar development is different from that seen in other crustaceans (e.g., Cephalocarida and Branchiopoda: Anostraca) in which the postmandibular limbs in general appear gradually, a pattern generally considered plesiomorphic for “Crustacea” (=Pancrustacea) and also present in the Cambrian *Rehbachiella kinnekullensis* (Walossek 1993; Martin et al. 2014; Olesen 2018). It should be noted, however, that subgroups of Branchiopoda have much modified/accelerated larval development, even occasionally with suppressed postmandibular limbs (early notostracan larvae), but which are assumed to have evolved secondarily within Branchiopoda (Olesen and Martin 2014; Olesen and Møller 2014; Olesen 2014). All in all, and deviating from Oakley et al. (2013), we consider a shared presence of six naupliar larvae (=suppressed postmandibular limbs in early larval phase) in Thecostraca and Copepoda (=Hexanauplia) as a highly uncertain synapomorphy for these taxa, since malacostracan free-living nauplii can be argued to exhibit limb suppression in early development not very different from that seen in copepods. If anything, the mentioned limb suppression leading to the presence of a distinct naupliar phase early in development could equally well qualify as a synapomorphy for a clade composed of Thecostraca, Copepoda, and Malacostraca (=Multicrustacea sensu Regier et al. 2010). However, since the two malacostracan taxa with free nauplii (krill and dendrobranchiate shrimps) are never placed basally in malacostracan phylogeny, multiple loses of free nauplii within Malacostraca has to be assumed for them to represent an ancestral mode of development within Malacostraca (e.g., Scholtz 2000; Schwentner et al. 2018).

As outlined earlier, some crustaceans (Cephalocarida, Branchiopoda, and Remipedia) are well-known to have a gradual (anamorphic) development traditionally considered plesiomorphic for Crustacea (Sanders 1963; Walossek 1993). It is striking that taxa with such a development are placed in Allotriocarida close to Hexapoda (fig. 2). It is yet uncertain how to interpret this, but it may indicate that an extreme type of gradual development, with one somite added per moult, appeared after Allotriocarida split from Multicrustacea. Alternatively, such anamorphic development was present in the common ancestor to Altocrustacea since development in Copepoda, Thecostraca, and Malacostraca bears many traces of anamorphic development (regardless of limb suppression leading to presence of a naupliar phase).

## Conclusions

We have built two molecular matrices based on different orthology assignment strategies and shown that the matrix based on the selection of single-copy and slowly evolving genes is less affected by substitution saturation, previously found as a major confounding factor in deep-phylogenetic studies. Furthermore, although we always retrieve fully resolved trees, parts of the topologies depended heavily on the specific model used, suggesting weak phylogenetic signal in parts of the phylogeny. Strong support was found for a basal split in Pancrustacea between Oligostraca (Mystacocarida, Ostracoda, Ichthyostraca) and Altocrustacea (the remaining pancrustaceans), and for many classical groups such as Branchiopoda, Malacostraca, Copepoda, Remipedia, and Hexapoda. The addition of three newly sequenced remipedes confirmed the sister group relationship between Remipedia and Hexapoda (Labiocarida). We recovered Branchiopoda as the sister of Labiocarida, a clade for which we suggest the name Athalassocarida recognizing that its living members (remipedes, branchiopods, and hexapods) are either nonmarine or have secondarily reverted to marine environments. Within Allotriocarida Cephalocarida was sister to Athalassocarida (Branchiopoda, Remipedia, and Hexapoda). Moderate support was found for Hexanauplia (Copepoda sister to Thecostraca) in alliance with Malacostraca in a Multicrustacea clade, without completely rejecting a possible sister-group relationship between Thecostraca and Malacostraca. We found the position of Copepoda to be very sensitive to changes in analytical approach and suggest that a more complete taxon sampling of this particular lineage will be needed to robustly assess their phylogenetic position. Based on superimposing key crustacean tagmosis and developmental patterns over the most robust phylogeny, we hypothesize that the ancestral condition of Pancrustacea was characterized by a relatively short body, and that extreme body elongation and possibly anamorphic development evolved later in the evolution of the group.

## Supplementary Material

Supplementary data are available at *Genome Biology and Evolution* online.

## Supplementary Material

Supplementary_Data_evz097

## Notes

**Data deposition:** This project has been deposited at the NCBI under the accession number PRJNA507978 (SRA numbers: SRR8280776/SRR8280777/SRR8280778).
