# Supplementary material for: Pancrustacean Evolution Illuminated by Taxon-Rich Genomic-Scale Data Sets with an Expanded Remipede Sampling
Source: Genome Biol Evol. 2019 Jul 4;11(8):2055–70. doi: 10.1093/gbe/evz097 (PMC6684935; doi:10.1093/gbe/evz097)
Supplement: Supplementary_Data_evz097 [file supplementary_data_evz097.pdf]

Supplementary Information

Pancrustacean evolution illuminated by taxon-rich genomic-scale data sets with an expanded remipede sampling

Jesus Lozano-Fernandez, Mattia Giacomelli, James Fleming, Albert Chen, Jakob Vinther, Philip Francis Thomsen, Henrik Glenner, Ferran Palero, David A. Legg, Thomas M. Iliffe, Davide Pisani, Jørgen Olesen

Table of contents:

Supplementary Figure 1 2

Supplementary Figure 2 3-6

Supplementary Table 1 7-9

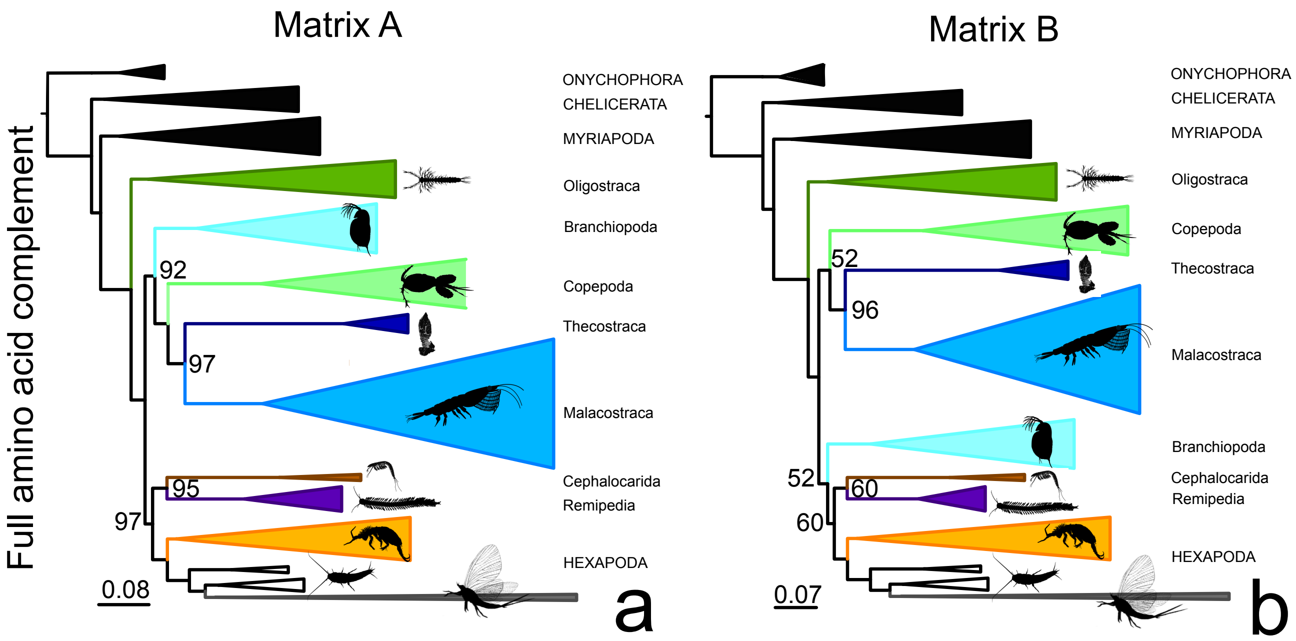

Figure S1. Schematic representations of the relationships inferred using ML under the LG+I+G4 model **(A)** on Matrix A **(B)** and on Matrix B. Support values represent bootstrap support and only those below 100 are shown.

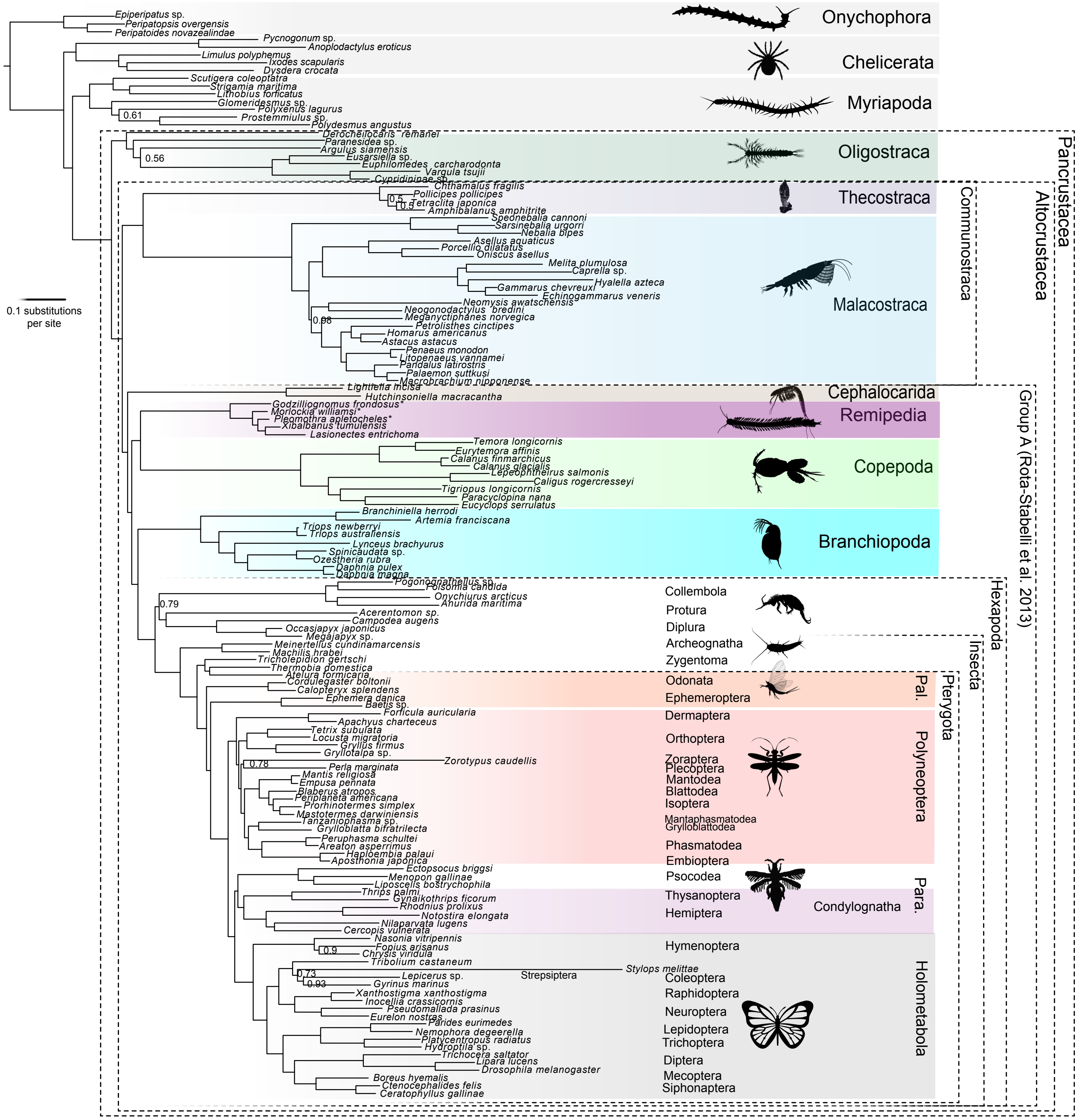

Epiperipatus sp.
Peripatopsis overgensis
Peripatoides novazealindae
Onychophora
Pycnogonum sp.
Anoplodactylus eroticus
Limulus polyphemus
Ixodes scapularis
Dysdera crocata
Chelicerata
Scutigera coleoptrata
Strigamia maritima
Lithobius forficatus
Glomeridesmus sp.
Polyxenus lagurus
0.61
Prostemmiulus sp.
Polydesmus angustus
Myriapoda
0.1 substitutions per site
Derocheilocaris remanei
Paranesidea sp.
Argulus siamensis
0.56
Eusarsiella sp.
Euphilomedes carcharodonta
Vargula tsujii
Cypridininae sp.
Oligostraca
Chthamalus fragilis
Pollicipes pollicipes
0.5
Tetraclita japonica
0.5
Amphibalanus amphitrite
Thecostraca
Speonebalia cannoni
Sarsinebalia urgorri
Nebalia bipes
Asellus aquaticus
Porcellio dilatatus
Oniscus asellus
Melita plumulosa
Caprella sp.
Hyalella azteca
Gammarus chevreuxi
Echinogammarus veneris
Neomysis awatschensis
Neogonodactylus bredini
0.98
Meganyctiphanes norvegica
Petrolisthes cinctipes
Homarus americanus
Astacus astacus
Penaeus monodon
Litopenaeus vannamei
Pandalus latirostris
Palaemon suttkusi
Macrobrachium nipponense
Malacostraca
Communostraca
Altocrustacea
Pancrustacea
Lightiella incisa
Hutchinsoniella macracantha
Cephalocarida
Godzilliognomus frondosus*
Morlockia williamsi*
Pleomothra apletocheles*
Xibalbanus tumulensis
Lasionectes entrichoma
Remipedia
Temora longicornis
Eurytemora affinis
Calanus finmarchicus
Calanus glacialis
Lepeophtheirus salmonis
Caligus rogercresseyi
Tigriopus longicornis
Paracyclopina nana
Eucyclops serrulatus
Copepoda
Branchiniella herrodi
Artemia franciscana
Triops newberryi
Triops australiensis
Lynceus brachyurus
Spinicaudata sp.
Ozestheria rubra
Daphnia pulex
Daphnia magna
Branchiopoda
Group A (Rota-Stabelli et al. 2013)
Pogonognathellus sp.
Folsomia candida
Onychiurus arcticus
Anurida maritima
0.79
Acerentomon sp.
Campodea augens
Occasjapyx japonicus
Megajapyx sp.
Meinertellus cundinamarcensis
Machilis hrabei
Tricholepidion gertschi
Thermobia domestica
Atelura formicaria
Cordulegaster boltonii
Calopteryx splendens
Ephemera danica
Baetis sp.
Collembola
Protura
Diplura
Archeognatha
Zygentoma
Odonata
Ephemeroptera
Pal.
Hexapoda
Insecta
Pterygota
Forficula auricularia
Apachyus charteceus
Tetrix subulata
Locusta migratoria
Gryllus firmus
Gryllotalpa sp.
Zorotypus caudellis
0.78
Perla marginata
Mantis religiosa
Empusa pennata
Blaberus atropos
Periplaneta americana
Prorhinotermes simplex
Mastotermes darwiniensis
Tanzaniophasma sp.
Grylloblatta bifratrilecta
Peruphasma schultei
Areaton asperrimus
Haploembia palaui
Aposthonia japonica
Dermaptera
Orthoptera
Zoraptera
Plecoptera
Mantodea
Blattodea
Isoptera
Mantaphasmatodea
Grylloblattodea
Phasmatodea
Embioptera
Polyneoptera
Ectopsocus briggsi
Menopon gallinae
Liposcelis bostrychophila
Thrips palmi
Gynaikothrips ficorum
Rhodnius prolixus
Notostira elongata
Nilaparvata lugens
Cercopis vulnerata
Psocodea
Thysanoptera
Hemiptera
Condylognatha
Para.
Nasonia vitripennis
Fopius arisanus
0.9
Chrysis viridula
Tribolium castaneum
Stylops melittae
Strepsiptera
0.73
Lepicerus sp.
0.93
Gyrinus marinus
Xanthostigma xanthostigma
Inocellia crassicornis
Pseudomallada prasinus
Eurelon nostras
Parides eurimedes
Nemophora degeerella
Platycentropus radiatus
Hydroptila sp.
Trichocera saltator
Lipara lucens
Drosophila melanogaster
Boreus hyemalis
Ctenocephalides felis
Ceratophyllus gallinae
Hymenoptera
Coleoptera
Raphidoptera
Neuroptera
Lepidoptera
Trichoptera
Diptera
Mecoptera
Siphonaptera
Holometabola

a

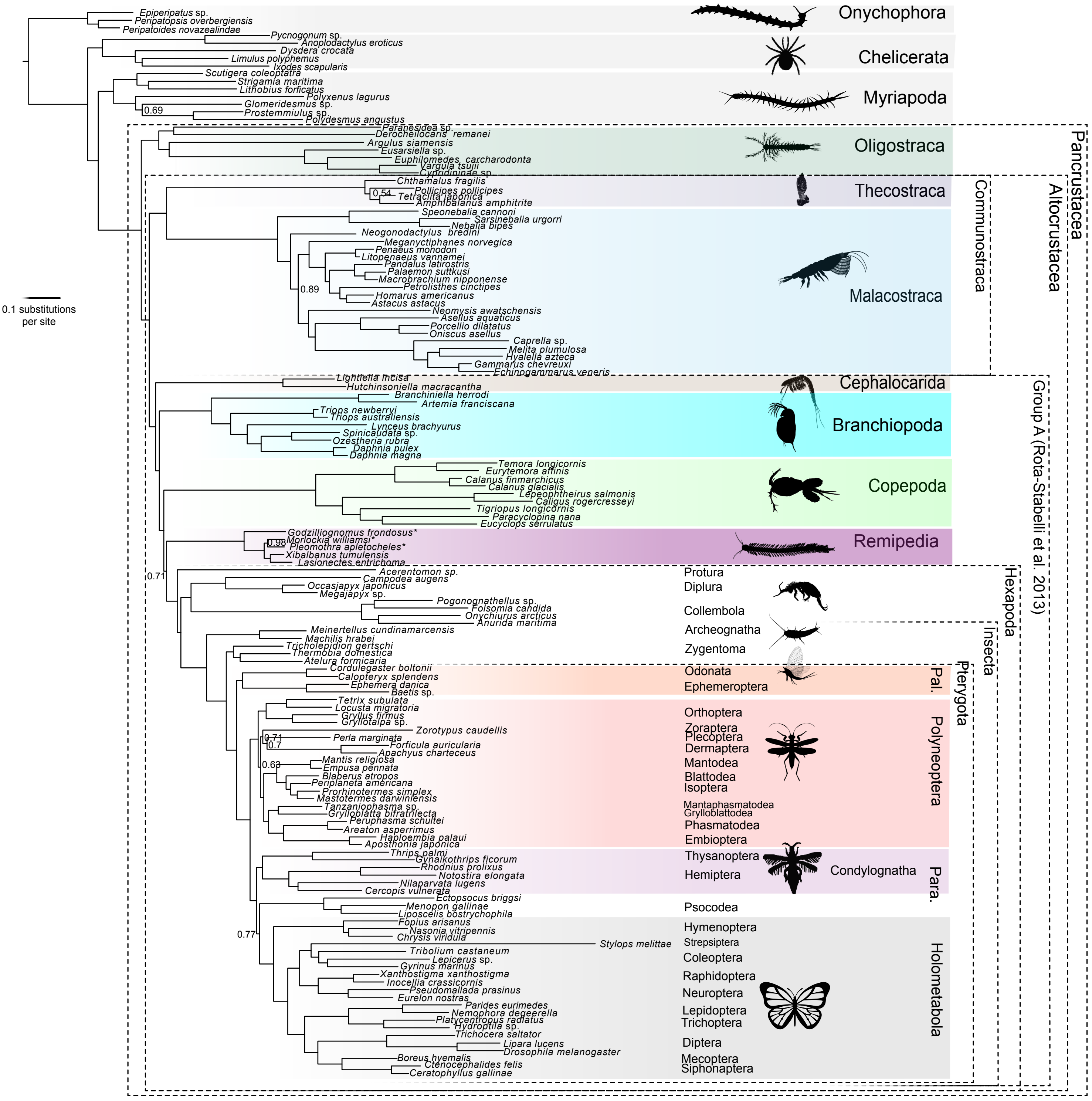

Epiperipatus sp.
Peripatopsis overbergiensis
Peripatoides novazealindae
Pycnogonum sp.
Anoplodactylus eroticus
Dysdera crocata
Limulus polyphemus
Ixodes scapularis
Scutigera coleoptrata
Strigamia maritima
Lithobius forficatus
Polyxenus lagurus
Glomeridesmus sp.
Prostemmiulus sp.
Polydesmus angustus
0.69
Onychophora
Chelicerata
Myriapoda
0.1 substitutions per site
Paranesidea sp.
Derocheilocaris remanei
Argulus siamensis
Eusarsiella sp.
Euphilomedes carcharodonta
Vargula tsujii
Cypridininae sp.
Oligostraca
Chthamalus fragilis
Pollicipes pollicipes
Tetraclita japonica
Amphibalanus amphitrite
0.54
Thecostraca
Speonebalia cannoni
Sarsinebalia urgorri
Nebalia bipes
Neogonodactylus bredini
Meganyctiphanes norvegica
Penaeus monodon
Litopenaeus vannamei
Pandalus latirostris
Palaemon suttkusi
Macrobrachium nipponense
Petrolisthes cinctipes
Homarus americanus
Astacus astacus
0.89
Neomysis awatschensis
Asellus aquaticus
Porcellio dilatatus
Oniscus asellus
Caprella sp.
Melita plumulosa
Hyalella azteca
Gammarus chevreuxi
Echinogammarus veneris
Malacostraca
Communostraca
Altocrustacea
Pancrustacea
Lightiella incisa
Hutchinsoniella macracantha
Cephalocarida
Branchinella herrodi
Artemia franciscana
Triops newberryi
Thops australiensis
Lynceus brachyurus
Spinicaudata sp.
Ozestheria rubra
Daphnia pulex
Daphnia magna
Branchiopoda
Temora longicornis
Eurytemora affinis
Calanus finmarchicus
Calanus glacialis
Lepeophtheirus salmonis
Caligus rogercresseyi
Tigriopus longicornis
Paracyclopina nana
Eucyclops serrulatus
Copepoda
Godzilliognomus frondosus*
Morlockia williamsi*
Pleomothra apletocheles*
Xibalbanus tumulensis
Lasionectes entrichoma
0.98
Remipedia
Group A (Rota-Stabelli et al. 2013)
0.71
Acerentomon sp.
Campodea augens
Occasjapyx japonicus
Megajapyx sp.
Pogonognathellus sp.
Folsomia candida
Onychiurus arcticus
Anurida maritima
Protura
Diplura
Collembola
Meinertellus cundinamarcensis
Machilis hrabei
Tricholepidion gertschi
Thermobia domestica
Atelura formicaria
Archeognatha
Zygentoma
Hexapoda
Insecta
Cordulegaster boltonii
Calopteryx splendens
Ephemera danica
Baetis sp.
Odonata
Ephemeroptera
Pal.
Pterygota
Tetrix subulata
Locusta migratoria
Gryllus firmus
Gryllotalpa sp.
Zorotypus caudellis
Perla marginata
Forficula auricularia
Apachyus charteceus
Mantis religiosa
Empusa pennata
Blaberus atropos
Periplaneta americana
Prorhinotermes simplex
Mastotermes darwiniensis
Tanzaniophasma sp.
Grylloblatta bifratrilecta
Peruphasma schultei
Areaton asperrimus
Haploembia palaui
Aposthonia japonica
0.71
0.7
0.68
Orthoptera
Zoraptera
Plecoptera
Dermaptera
Mantodea
Blattodea
Isoptera
Mantaphasmatodea
Grylloblattodea
Phasmatodea
Embioptera
Polyneoptera
Thrips palmi
Gynaikothrips ficorum
Rhodnius prolixus
Notostira elongata
Nilaparvata lugens
Cercopis vulnerata
Thysanoptera
Hemiptera
Condylognatha
Para.
Ectopsocus briggsi
Menopon gallinae
Liposcelis bostrychophila
Psocodea
0.77
Fopius arisanus
Nasonia vitripennis
Chrysis viridula
Stylops melittae
Tribolium castaneum
Lepicerus sp.
Gyrinus marinus
Xanthostigma xanthostigma
Inocellia crassicornis
Pseudomallada prasinus
Eurelon nostras
Parides eurimedes
Nemophora degeerella
Platycentropus radiatus
Hydroptila sp.
Trichocera saltator
Lipara lucens
Drosophila melanogaster
Boreus hyemalis
Ctenocephalides felis
Ceratophyllus gallinae
Hymenoptera
Strepsiptera
Coleoptera
Raphidoptera
Neuroptera
Lepidoptera
Trichoptera
Diptera
Mecoptera
Siphonaptera
Holometabola

b

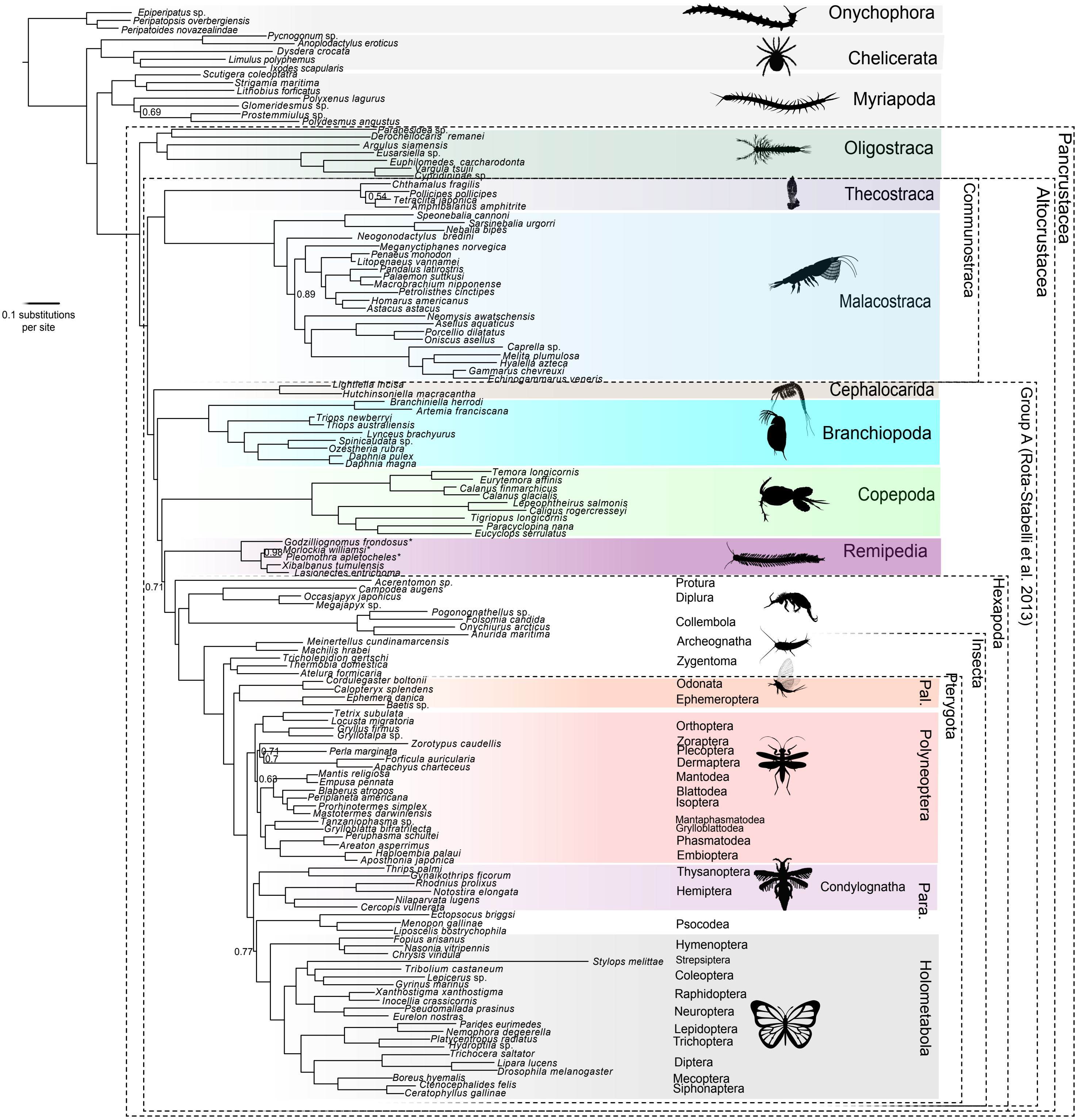
Onychophora
Chelicerata
Myriapoda
Oligostraca
Thecostraca
Malacostraca
Cephalocarida
Branchiopoda
Copepoda
Remipedia
Pancrustacea
Altocrustacea
Communostraca
Group A (Rota-Stabelli et al. 2013)
Hexapoda
Insecta
Pterygota
Pal.
Polyneoptera
Para.
Holometabola
Condylognatha
0.1 substitutions per site
Epiperipatus sp.
Peripatopsis overbergiensis
Peripatoides novazealindae
Pycnogonum sp.
Anoplodactylus eroticus
Dysdera crocata
Limulus polyphemus
Ixodes scapularis
Scutigera coleoptrata
Strigamia maritima
Lithobius forficatus
Polyxenus lagurus
Glomeridesmus sp.
0.69
Prostemmiulus sp.
Polydesmus angustus
Paranesidea sp.
Derocheilocaris remanei
Argulus siamensis
Eusarsiella sp.
Euphilomedes carcharodonta
Vargula tsujii
Cypridininae sp.
Chthamalus fragilis
Pollicipes pollicipes
0.54
Tetraclita japonica
Amphibalanus amphitrite
Speonebalia cannoni
Sarsinebalia urgorri
Nebalia bipes
Neogonodactylus bredini
Meganyctiphanes norvegica
Penaeus monodon
Litopenaeus vannamei
Pandalus latirostris
Palaemon suttkusi
Macrobrachium nipponense
Petrolisthes cinctipes
0.89
Homarus americanus
Astacus astacus
Neomysis awatschensis
Asellus aquaticus
Porcellio dilatatus
Oniscus asellus
Caprella sp.
Melita plumulosa
Hyalella azteca
Gammarus chevreuxi
Echinogammarus veneris
Lightiella incisa
Hutchinsoniella macracantha
Branchiniella herrodi
Artemia franciscana
Triops newberryi
Triops australiensis
Lynceus brachyurus
Spinicaudata sp.
Ozestheria rubra
Daphnia pulex
Daphnia magna
Temora longicornis
Eurytemora affinis
Calanus finmarchicus
Calanus glacialis
Lepeophtheirus salmonis
Caligus rogercresseyi
Tigriopus longicornis
Paracyclopina nana
Eucyclops serrulatus
Godzilliognomus frondosus*
0.98
Morlockia williamsi*
Pleomothra apletocheles*
Xibalbanus tumulensis
Lasionectes entrichoma
0.71
Acerentomon sp.
Campodea augens
Occasjapyx japonicus
Megajapyx sp.
Pogonognathellus sp.
Folsomia candida
Onychiurus arcticus
Anurida maritima
Meinertellus cundinamarcensis
Machilis hrabei
Tricholepidion gertschi
Thermobia domestica
Atelura formicaria
Cordulegaster boltonii
Calopteryx splendens
Ephemera danica
Baetis sp.
Tetrix subulata
Locusta migratoria
Gryllus firmus
Gryllotalpa sp.
Zorotypus caudellis
0.71
Perla marginata
0.7
Forficula auricularia
Apachyus charteceus
Mantis religiosa
0.63
Empusa pennata
Blaberus atropos
Periplaneta americana
Prorhinotermes simplex
Mastotermes darwiniensis
Tanzaniophasma sp.
Grylloblatta bifratrilecta
Peruphasma schultei
Areaton asperrimus
Haploembia palaui
Aposthonia japonica
Thrips palmi
Gynaikothrips ficorum
Rhodnius prolixus
Notostira elongata
Nilaparvata lugens
Cercopis vulnerata
Ectopsocus briggsi
Menopon gallinae
Liposcelis bostrychophila
0.77
Fopius arisanus
Nasonia vitripennis
Chrysis viridula
Stylops melittae
Tribolium castaneum
Lepicerus sp.
Gyrinus marinus
Xanthostigma xanthostigma
Inocellia crassicornis
Pseudomallada prasinus
Eurelon nostras
Parides eurimedes
Nemophora degeerella
Platycentropus radiatus
Hydroptila sp.
Trichocera saltator
Lipara lucens
Drosophila melanogaster
Boreus hyemalis
Ctenocephalides felis
Ceratophyllus gallinae
Protura
Diplura
Collembola
Archeognatha
Zygentoma
Odonata
Ephemeroptera
Orthoptera
Zoraptera
Plecoptera
Dermaptera
Mantodea
Blattodea
Isoptera
Mantaphasmatodea
Grylloblattodea
Phasmatodea
Embioptera
Thysanoptera
Hemiptera
Psocodea
Hymenoptera
Strepsiptera
Coleoptera
Raphidoptera
Neuroptera
Lepidoptera
Trichoptera
Diptera
Mecoptera
Siphonaptera

C

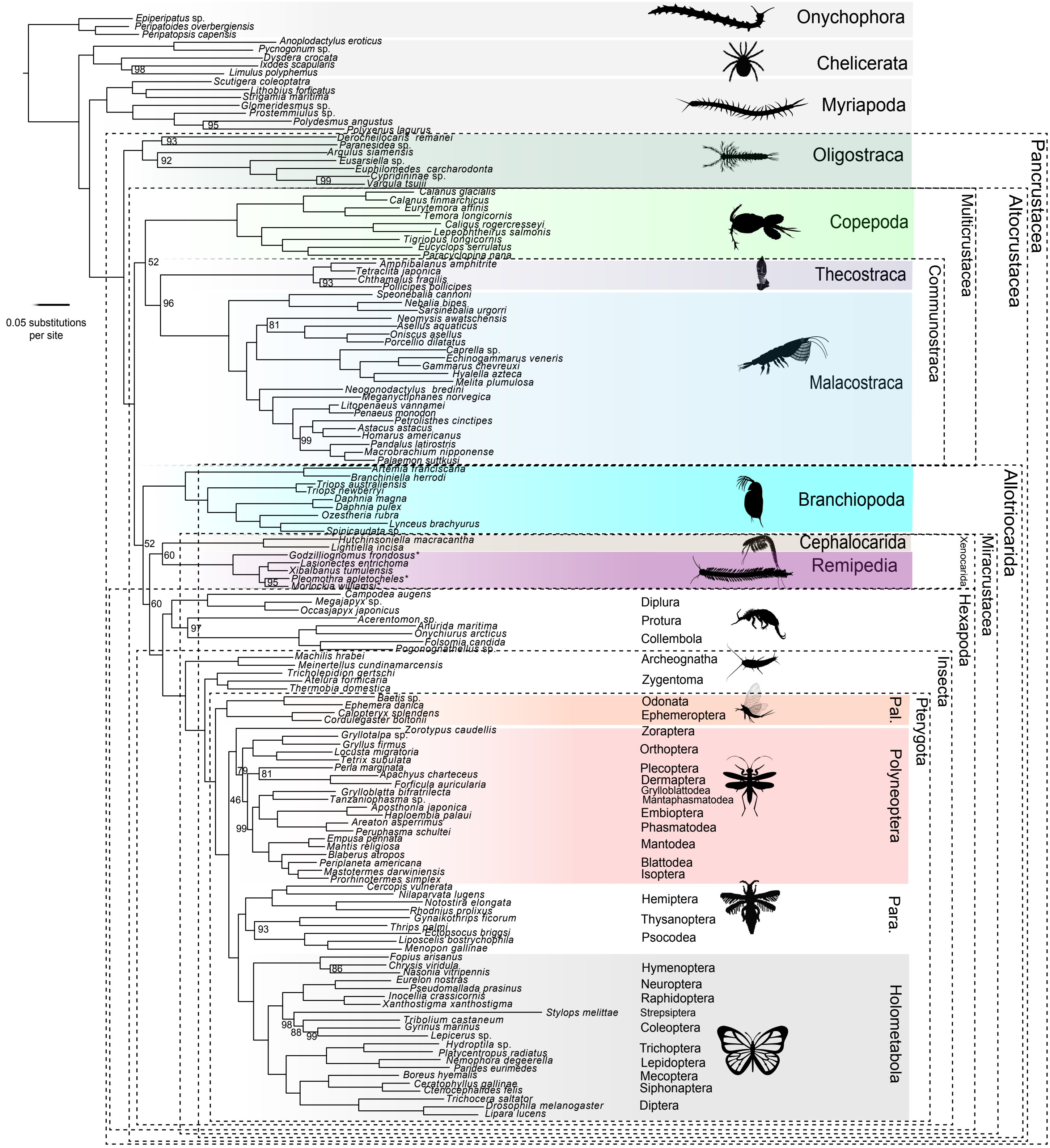

d

Supplementary Figure 2. Detailed phylogenetic trees: Bayesian analyses of **(A)** CAT-GTR+G analysis of Matrix A **(B)** and CAT-GTR+G analysis of Matrix B, and ML analyses of **(C)** Matrix A analysed under the LG+I+G4 model **(D)** and Matrix B analysed under the LG+I+G4 model. Support values represent posterior probabilities **(A-B)** or bootstrap support **(C-D)** and only those below 1 or below 100, Bayesian and ML respectively, are shown. Newly sequenced remipede transcriptomes marked with an asterisk.

Table S1. Species names and accession numbers from the species used in this study.

Class/Clade	Species	Accession
**Crustacea: Branchiopoda**	*Artemia franciscana-GA17E*	SRR1324814
**Crustacea: Branchiopoda**	*Branchinella herrodi*	SRR4113492
**Crustacea: Branchiopoda**	***Daphnia magna***	GDIP00000000.1
**Crustacea: Branchiopoda**	*Daphnia pulex*	PRJNA12756
**Crustacea: Branchiopoda**	*Lynceus brachyurus*	67305[BioProject]
**Crustacea: Branchiopoda**	*Ozestheria rubra*	SRR4113504
**Crustacea: Branchiopoda**	*Spinicaudata sp. BMR-2011*	67311[BioProject]
**Crustacea: Branchiopoda**	*Triops australiensis*	SRR4113493
**Crustacea: Branchiopoda**	***Triops newberryi***	GEHY00000000.1
**Crustacea: Cephalocarida**	***Hutchinsoniella macracantha***	SRR4113505
**Crustacea: Cephalocarida**	*Lightiella incisa*	SRR4113506
**Crustacea: Copepoda**	*Calanus finmarchicus*	SRR4113507
**Crustacea: Copepoda**	*Calanus glacialis*	HACJ00000000
**Crustacea: Copepoda**	*Caligus rogercresseyi*	GAZX00000000
**Crustacea: Copepoda**	*Eucyclops serrulatus*	GARW00000000.1
**Crustacea: Copepoda**	***Eurytemora affinis***	GEAN00000000.1
**Crustacea: Copepoda**	*Lepeophtheirus salmonis*	HACA00000000
**Crustacea: Copepoda**	*Paracyclopina nana*	GCJT00000000
**Crustacea: Copepoda**	*Temora longicornis*	SRR4113508
**Crustacea: Copepoda**	***Tigriopus japonicus***	GCHA00000000.1
**Crustacea: Malacostraca:Peracarida:Isopoda**	*Asellus aquaticus*	GDKY00000000.1
**Crustacea: Malacostraca:Eucarida:Decapoda:Pleocyemata**	***Astacus astacus***	GEDF01000000.1
**Crustacea: Malacostraca:Peracarida:Amphipoda**	*Caprella sp.*	SRR4113494
**Crustacea: Malacostraca:Peracarida:Amphipoda**	***Echinogammarus veneris***	GARO00000000.1
**Crustacea: Malacostraca:Peracarida:Amphipoda**	*Gammarus chevreuxi*	GFCV00000000.1
**Crustacea: Malacostraca:Eucarida:Decapoda**	*Homarus americanus*	GEBG00000000
**Crustacea: Malacostraca:Peracarida:Amphipoda**	***Hyalella azteca***	GEHV00000000.1
**Crustacea: Malacostraca:Eucarida:Decapoda:Dendrobranchiata**	*Litopenaeus vannamei*	GETZ00000000
**Crustacea: Malacostraca:Eucarida:Decapoda:Pleocyemata**	*Macrobrachium nipponense*	GCVG00000000.1
**Crustacea: Malacostraca:Eucarida:Euphausiacea**	***Meganyctiphanes norvegica***	GETT00000000.1
**Crustacea: Malacostraca:Peracarida:Amphipoda**	*Melita plumulosa*	GAKD00000000.1
**Crustacea: Malacostraca:Peracarida:Isopoda**	*Oniscus asellus*	SRR3263253
**Crustacea: Malacostraca:Phyllocarida:Leptostraca**	*Nebalia bipes*	PRJNA67309
**Crustacea: Malacostraca:Eumalacostraca:Hoplocarida:Stomatopoda**	*Neogonodactylus bredini*	SRR4113511
**Crustacea: Malacostraca:Peracarida:Mysidacea**	*Neomysis awatschensis*	GDFV00000000.1
**Crustacea: Malacostraca:Eucarida:Decapoda:Pleocyemata**	*Palaemon suttkusi*	SRR4113510
**Crustacea: Malacostraca:Eumalacostraca:Decapoda:Pleocyemata**	*Pandalus latirostris*	DRR001118
**Crustacea: Malacostraca:Eucarida:Decapoda:Dendrobranchiata**	*Penaeus monodon*	GFCA00000000
**Crustacea: Malacostraca:Eucarida:Decapoda:Pleocyemata**	*Petrolisthes cinctipes*	EST NCBI
**Crustacea: Malacostraca:Eumalacostraca:Peracarida:Isopoda**	*Porcellio dilatatus*	SRR5253654
**Crustacea: Malacostraca:Phyllocarida:Leptostraca**	*Sarsinebalia urgorrii*	66993[BioProject]
**Crustacea: Malacostraca:Phyllocarida:Leptostraca**	*Speonebalia cannoni*	SRR4113509
**Crustacea: Mystacocarida**	***Derocheilocaris remanei***	SRR4113503
**Crustacea: Oligostraca**	***Argulus siamensis***	SRR514120
**Crustacea: Oligostraca**	*Cypridininae sp.*	PRJNA66997

**Crustacea: Oligostraca**	*Euphilomedes carcharodonta*	SRR2102714
**Crustacea: Ostracoda**	*Eusarsiella sp.*	SRR4113497
**Crustacea: Ostracoda**	*Paranesidea sp.*	SRR4113496
**Crustacea: Ostracoda**	*Vargula tsujii*	SRR1269674
**Crustacea: Remipedia**	*Godzilliognomus* cf. *frondosus*	SRR8280777
**Crustacea: Remipedia**	*Lasionectes entrichoma*	SRR4113498
**Crustacea: Remipedia**	*Pleomothra apletocheles*	SRR8280776
**Crustacea: Remipedia**	*Morlockia williamsi*	SRR8280778
**Crustacea: Remipedia**	***Xibalbanus tulumensis***	SRR4113501
**Crustacea: Thecostraca**	*Amphibalanus amphitrite*	SRR2034994
**Crustacea: Thecostraca**	*Chthamalus fragilis*	SRR4113502
**Crustacea: Thecostraca**	*Pollicipes pollicipes*	EST NCBI
**Crustacea: Thecostraca**	*Tetraclita japonica*	SRR426837
**Hexapoda: Archaeognatha**	***Machilis hrabei***	GAUM00000000.2
**Hexapoda: Archaeognatha**	*Meinertellus cundinamarcensis*	GAUG00000000.2
**Hexapoda: Collembola**	***Anurida maritima***	GAUE00000000.2
**Hexapoda: Collembola**	***Folsomia candida***	GASX00000000.2
**Hexapoda: Collembola**	*Onychiurus Arcticus*	EST NCBI
**Hexapoda: Collembola**	*Pogonognathellus sp. AD-2013*	GATD00000000.2
**Hexapoda: Diplura**	***Campodea augens***	GAYN00000000.2
**Hexapoda: Diplura**	*Megajapyx sp.*	SRR400673
**Hexapoda: Diplura**	*Occasjapyx japonicus*	GAXJ00000000.2
**Hexapoda: Protura**	***Acerentomon sp.***	GAXE00000000.2
**Hexapoda: Zygentoma**	***Atelura formicaria***	GAYJ00000000.2
**Hexapoda: Zygentoma**	*Thermobia domestica*	GASN00000000.2
**Hexapoda: Zygentoma**	*Tricholepidion gertschi*	GASO00000000
**Insect: Blattodea**	***Blaberus atropos***	GAYD00000000.2
**Insect: Blattodea**	***Periplaneta americana***	GAWS00000000.2
**Insect: Coleoptera**	***Gyrinus marinus***	GAUY00000000.2
**Insect: Coleoptera**	***Lepicerus sp. AD-2013***	GAZB00000000.2
**Insect: Coleoptera**	*Tribolium castaneum*	PRJNA12540
**Insect: Dermaptera**	*Apachyus charteceus*	GAUW00000000.2
**Insect: Dermaptera**	***Forficula auricularia***	GAYQ00000000.2
**Insect: Diptera**	*Drosophila melanogaster*	PRJNA13812
**Insect: Diptera**	*Lipara lucens*	GAZD00000000.2
**Insect: Diptera**	***Trichocera saltator***	GAXZ00000000.2
**Insect: Embioptera**	***Aposthonia japonica***	GAWU00000000.2
**Insect: Embioptera**	***Haploembia palaui***	GAZA00000000.2
**Insect: Ephemeroptera**	***Baetis sp.***	GATU00000000.2
**Insect: Ephemeroptera**	***Ephemera danica***	GAUK00000000.2
**Insect: Grylloblattidae**	***Grylloblatta bifratrilecta***	GAWP00000000.2
**Insect: Hemiptera**	***Cercopis vulnerata***	GAUN00000000.2
**Insect: Hemiptera**	***Nilaparvata lugens***	GAYF00000000.2
**Insect: Hemiptera**	*Notostira elongata*	GASV00000000.2
**Insect: Hemiptera**	*Rhodnius prolixus*	GECK00000000
**Insect: Hymenoptera**	***Chrysis viridula***	GATY00000000.2
**Insect: Hymenoptera**	***Fopius arisanus***	GBYB00000000.1
**Insect: Hymenoptera**	*Nasonia vitripennis*	GBEB00000000
**Insect: Isoptera**	***Mastotermes darwiniensis***	GAZE00000000.2

**Insect: Isoptera**	*Prorhinotermes simplex*	GASE00000000.2
**Insect: Lepidoptera**	*Nemophora degeerella*	GATC00000000.2
**Insect: Lepidoptera**	***Parides eurimedes***	GAXH00000000.2
**Insect: Mantodea**	***Empusa pennata***	GAWT00000000.2
**Insect: Mantodea**	***Mantis religiosa***	GASW00000000.2
**Insect: Mantophasmatodea**	***Tanzaniophasma sp. AD-2013***	GAXB00000000.
**Insect: Mecoptera**	***Boreus hyemalis***	GAYK00000000.2
**Insect: Neuroptera**	***Pseudomallada prasinus***	GAVV00000000.2
**Insect: Odonata**	***Calopteryx splendens***	GAYM00000000.2
**Insect: Odonata**	***Cordulegaster boltonii***	GAYO00000000.2
**Insect: Orthoptera**	***Gryllotalpa sp.***	GAWZ00000000.2
**Insect: Orthoptera**	*Gryllus firmus*	GAIZ00000000
**Insect: Orthoptera**	*Locusta migratoria*	SRR2037099
**Insect: Orthoptera**	*Tetrix subulata*	GASQ00000000.2
**Insect: Phasmatodea**	*Aretaon asperrimus*	GAWC00000000.1
**Insect: Phasmatodea**	***Peruphasma schultei***	GAWJ00000000.2
**Insect: Phthiraptera**	***Menopon gallinae***	GAWR00000000.2
**Insect: Plecoptera**	***Perla marginata***	GATV00000000.2
**Insect: Psocodea**	*Liposcelis bostrychophila*	GAYV00000000.2
**Insect: Psocoptera**	***Ectopsocus briggsi***	GAPT00000000.2
**Insect: Raphidioptera**	***Inocellia crassicornis***	GAZH00000000.2
**Insect: Raphidioptera**	*Xanthostigma xanthostigma*	GAUI00000000.2
**Insect: Siphonaptera**	***Ceratophyllus gallinae***	GAWK00000000.2
**Insect: Siphonaptera**	***Ctenocephalides felis***	GAYP00000000.2
**Insect: Strepsiptera**	*Stylops melittae*	GAZM00000000.2
**Insect: Thysanoptera**	***Gynaikothrips ficorum***	GAXG00000000.2
**Insect: Thysanoptera**	*Thrips palmi*	GAXC00000000.2
**Insect: Trichoptera**	***Hydroptila sp. AD-2013***	GAVM00000000.2
**Insect: Trichoptera**	*Platycentropus radiatus*	GASS00000000.2
**Insect: Zoraptera**	*Zorotypus caudelli*	GAYA00000000.2
**Insect:Neuroptera**	***Euroleon nostras***	GAXW00000000.2
**Outgroup: Chelicerata**	*Anoplodactylus eroticus*	Sharma et al. 2014
**Outgroup: Chelicerata**	*Dysdera crocota*	SRR1328258
**Outgroup: Chelicerata**	***Ixodes scapularis***	SRR1189647
**Outgroup: Chelicerata**	*Limulus polyphemus*	SRR1145732
**Outgroup: Chelicerata**	*Pycnogonum sp.*	SRR8745912
**Outgroup: Myriapoda**	*Glomeridesmus sp.*	SRR941771
**Outgroup: Myriapoda**	*Lithobius forficatus*	GBKE00000000
**Outgroup: Myriapoda**	*Polydesmus angustus*	GBKG00000000
**Outgroup: Myriapoda**	*Polyxenus lagurus*	GBKF00000000
**Outgroup: Myriapoda**	*Prostemmiulus sp.*	SRR945439
**Outgroup: Myriapoda**	***Scutigera coleoptrata***	SRR1158078
**Outgroup: Myriapoda**	*Strigamia maritima*	SRR1267275
**Outgroup: Onychophora (Peripatidae)**	*Epiperipatus sp.*	Roeding, F., *et al.* 2007
**Outgroup: Onychophora (Peripatopsidae)**	*Peripatoides novaezealandiae*	SRR8745911
**Outgroup: Onychophora (Peripatopsidae)**	*Peripatopsis overbergiensis*	SRX451023

In **black** species used initially during the OMA orthology-assignment
